# *KCNV2*-Deficient Retinal Organoid Model of Cone Dystrophy—In Vitro Screening for AAV Gene Replacement Therapy

**DOI:** 10.3390/ijms27010449

**Published:** 2025-12-31

**Authors:** Sophie L. Busson, Arifa Naeem, Silvia Ferrara, Shilpita Sarcar, Toyin Adefila-Ideozu, Sarah Wells, Sophia El Alami, James Boot, Paul E. Sladen, Michel Michaelides, Anastasios Georgiadis, Amelia Lane

**Affiliations:** 1 MeiraGTx UK II, 34-38 Provost Street, London N1 7NG, UKmichel.michaelides@ucl.ac.uk (M.M.); tassos.georgiadis@meiragtx.com (A.G.); 2 Genome Centre, Blizard Institute, Queen Mary University of London, London E1 2AT, UK; 3 Moorfields Eye Hospital, 162 City Road, London EC1V 2PD, UK; 4 UCL Institute of Ophthalmology, University College London, London EC1V 9LF, UK

**Keywords:** *KCNV2*, inherited retinal disease, adeno-associated virus, gene therapy, IPSC, retinal organoids, CRISPR/Cas9, patient-derived, single-cell RNA sequencing, transcriptomics

## Abstract

*KCNV2* encodes Kv8.2, an electrically silent voltage-gated potassium channel subunit that is expressed in photoreceptors. Disease-causing variants in *KCNV2* cause a monogenic disorder which is classified clinically as cone dystrophy with supernormal rod response (CDSRR). Here, we generated *KCNV2*-deficient human retinal organoids as a tool for gene therapy vector potency assessment. The organoids were derived from two separate sources: by generating IPSCs from patient blood and by gene editing of a control cell line. Eight *KCNV2* gene therapy vectors were assessed in retinal organoids; Kv8.2 protein levels and its in situ interactions with potassium channel binding partners were quantitatively assessed. We show significant enhancements in vector potency and specificity by transgene codon optimisation and the use of the photoreceptor-specific rhodopsin kinase (RK) promoter, respectively. Single-cell RNA sequencing was performed in transduced retinal organoids to assess the performance of the AAV vectors at single-cell resolution. *KCNV2*-deficient photoreceptors had an upregulation in genes associated with apoptosis, oxidative stress, and hypoxia pathways which were partially restored in AAV-*KCNV2* transduced photoreceptors. These data show how human retinal organoids can be used to evaluate AAV gene therapy vector potency in vitro in a physiologically relevant model for the selection of lead therapeutic candidates and to help minimise the use of animals in preclinical development.

## 1. Introduction

*KCNV2* encodes Kv8.2, a modulatory ‘silent’ subunit which forms heteromeric channels with voltage-gated potassium channels in rod and cone photoreceptors [1]. Voltage-gated potassium channels in the retina produce a permanent outward current [2]. Compared to homomeric Kv channels, Kv8.2/Kv2 channels show a reduced inactivation from the open state. This is thought to allow a prolongation of opening in response to sustained depolarisations [3]. In the absence of Kv8.2, therefore, photoreceptors have impaired modulation of photoreceptor sensitivity in response to varying light levels. Disease-causing variants in *KCNV2* cause cone dystrophy with supernormal rod response (CDSRR), a rare, but severe, inherited retinal dystrophy with an autosomal recessive pattern of inheritance [4]. In a recent multicentre international clinical cohort study, 230 disease-causing alleles were identified, corresponding to 75 different *KCNV2* variants. The majority were missense variants (37%), followed by nonsense (24%) and frame-shifting indels (24%) with a cluster of missense variants in the highly conserved tetramerisation domain that facilitates interaction with Kv subunits [4]. The electroretinogram of affected individuals shows reduced rod and cone activity but a supernormal rod response to a bright flash of light [5] which is diagnostic for CDSRR. Notably, *KCNV2* retinopathy is relatively slow in progression, providing a large window for therapeutic intervention [6]. Importantly, the 1.6-kilobase (KB) *KCNV2* coding sequence and the required transgene expression regulatory elements are well within the 4.7 KB adeno-associated virus (AAV) packaging capacity. Retinal organoids (ROs) are an increasingly valuable part of the ophthalmological drug discovery toolkit [7]. In the last decade significant improvements in the efficiency of differentiation protocols has allowed for their production at a scale and quality that makes them a practical tool for drug development [8]. RO technology has proven particularly powerful in gene therapy development, where the potency and efficacy of both the transgene construct and AAV capsid can be interrogated in the context of the human retina [9,10,11,12,13,14,15,16]. ROs also enable the study of in situ protein–protein interactions in highly specialised and compartmentalised cells like photoreceptors.

Here, we developed a *Kv8.2*-deficient human RO model as a tool to assess the relative potency of eight novel AAV vectors designed to restore *KCNV2* protein expression to human photoreceptor cells. Interactions of the vector-derived protein with the potassium channel binding partners that are native to human photoreceptor cells were quantitively assessed and transcriptional responses to gene replacement in rod and cone photoreceptor cells were interrogated. These data demonstrate how ROs can be used to evaluate AAV gene therapy vector potency in vitro in the context of the human retina and to help minimise the use of animals in preclinical development.

## 2. Results

### 2.1. Potassium Channel Subunit KCNV2 Is Expressed During Retinal Organoid Development and Kv8.2 Localises to the Rod and Cone Inner Segments

**Figure 1:**
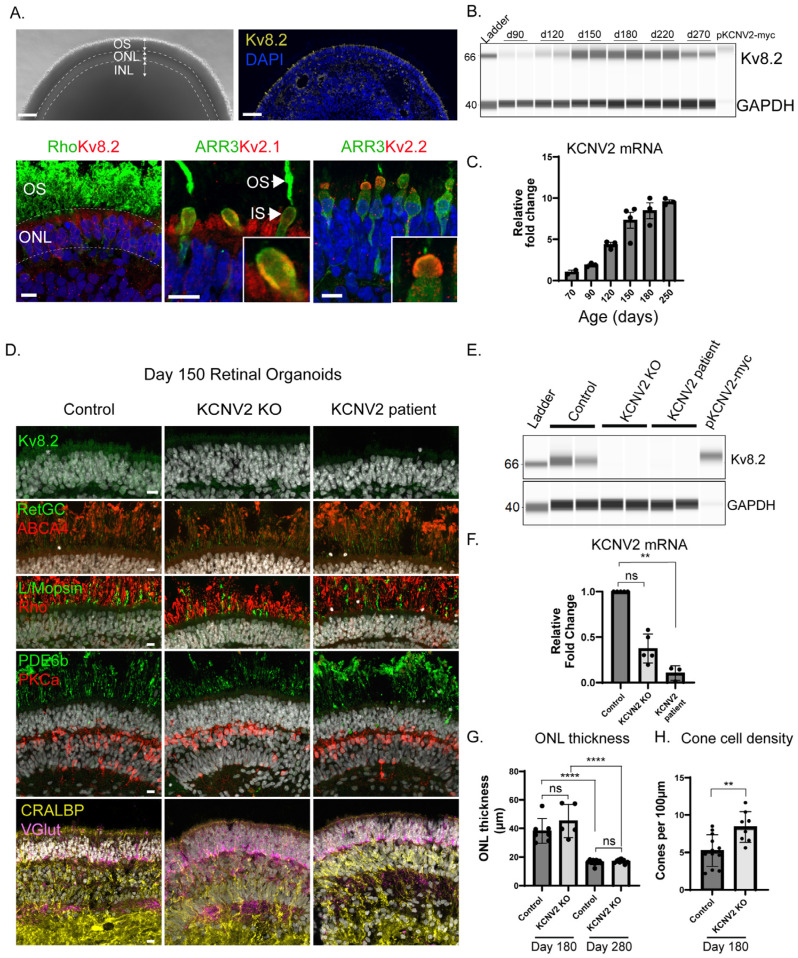
Voltage-gated Kv channel expression in control, *KCNV2* patient, and *KCNV2* CRISPR knockout retinal organoids. (**A**) Live imaging of a mature control retinal organoid with outer segments (OS) and distinctive photoreceptor outer nuclear layer (ONL). Low-magnification cryosection showing Kv8.2 expression (yellow) in outer nuclear layer. Kv8.2 (red; left panel) is diffusely expressed in rod and cone cell soma and inner segments but not the outer segments of day 160 ROs. Voltage-gated potassium channel Kv2.1 (red; middle panel) expression is restricted to the rod/cone inner segments (IS). Kv2.2 (red; right panel) expression is restricted to cone IS. Scale bars: top panels = 50 µm, bottom panels = 10 µm. INL = inner nuclear layer. (**B**) Wes analysis of Kv8.2 expression over a time course of control RO development. Each lane is a single organoid. MYC-tagged wt*KCNV2* plasmid transfected into HEK293 was included as a positive control. (**C**) Quantitative polymerase chain reaction (QPCR) for *KCNV2* mRNA over a time course of retinal development in control ROs. Each dot is an individual organoid, from a total of 3 separate differentiations. Fold change expressed relative to day 70. (**D**) Immunohistochemical (IHC) analysis for major retinal cell types in mature ROs derived from patient and *KCNV2* knockout (KO) and isogenic control organoids at day 150. Scale bar = 10 µm. (**E**) Kv8.2 protein in control, *KCNV2* KO, and patient ROs at day 90. HEK293 transfected with MYC-tagged wt*KCNV2* was included as a positive control. (**F**) QPCR for *KCNV2* mRNA in control, *KCNV2* KO, and patient ROs at day 180–190. Each dot is an individual RO. Fold change was calculated relative to control RO. ** *p* = 0.002 Kruskal–Wallis test, Dunn’s multiple comparisons test. ns = non-significant. (**G**) Mean ONL thickness quantification from 6 µm cryosections at day 180 and day 280 of control and *KCNV2* KO retinal organoids. Each dot represents a single organoid. There was no significant difference between cell lines at either time point but a significant difference between day 180 and day 280 in both cell lines. ANOVA; post hoc Tukey’s multiple comparison test: control d180 vs. *KCNV2* KO d180 ns *p* = 0.2370, control d180 vs. control d280 **** *p* < 0.0001, *KCNV2* KO d180 vs. *KCNV2* KO d280 **** *p* < 0.0001, control d280 vs. *KCNV2* KO d280 ns *p* = 0.9977. Control organoids *n* = 7 from *N* = 3 control differentiations, *KCNV2* KO *n* = 5 organoids from *N* = 4 differentiations, and d280 *n* = 10 organoids from *N* = 1 *KCNV2* KO and control differentiation. (**H**) Quantification of cones from L/M opsin staining in control and *KCNV2* KO organoids (data are from *N* = 6 *KCNV2* KO and *N* = 3 control independent differentiations). ** *p* = 0.004, Mann–Whitney test.

*KCNV2* expression and localisation were assessed in developing human retinal organoids derived from a control cell line with a functional *KCNV2* allele. In mature ROs, a distinctive photoreceptor layer, analogous to the outer nuclear layer (ONL) with outer segments (OS), was visible by live microscopy (Figure 1A—upper panel). Immunostaining of organoid cryosections at day 160 revealed expression of *KCNV2* protein product, Kv8.2, across this ONL layer but not in the rhodopsin (Rho)-positive OS (Figure 1A—lower panel). Voltage-gated potassium channels Kv2.1 and Kv2.2, with which Kv8.2 forms heteromers, were tightly confined to the photoreceptor inner segment (IS), whereas Kv8.2 expression appeared more diffuse across the ONL (Figure 1A—lower panel). Kv2.1 expression has previously been reported in rod and cone inner segments in human and macaque retina, whereas Kv2.2 is restricted to cone inner segments [1]. We observed the same cone-restricted distribution of Kv2.2 and rod/cone expression of Kv2.1 in control organoids (Figure 1A—lower panel).

Kv8.2 was detectable from whole-lysed organoids by automated capillary electrophoresis (Wes by ProteinSimple) immunoblotting from day 70–90 onward as single bands of 66 kDa (Figure 1B). *KCNV2* mRNA expression was detectable by differentiation at day 70 and increased on average 10-fold up to day 250 as the ROs continued to mature (Figure 1C).

### 2.2. Patient-Derived and CRISPR Knockout Retinal Organoid Model of KCNV2 Retinopathy

In order to test novel AAV vectors for transgene expression and function, *KCNV2* patient-derived induced pluripotent stem cells (IPSCs) were generated from peripheral blood monocyte cells (PBMCs). PBMCs were collected from a patient homozygous for a nonsense variant *KCNV2*: c.778A > T, p.(Lys260*). This was the third most common allele identified in an international clinical cohort study (7% of 230 alleles identified) [4]. IPSC lines from diverse genetic backgrounds can respond differently to retinal differentiation protocols, so, in addition to patient cells, CRISPR/Cas9 was used to establish an isogenic *KCNV2* knockout (KO) IPSC cell line via a simultaneous reprogramming and gene editing protocol [17] with three guide RNAs targeting *KCNV2* exon 1 (Supplementary Figure S1; Supplementary Table S5). ROs were derived from control IPSCs, patient IPSCs, and three separate CRISPR/Cas9 *KCNV2* KO IPSCs (hereafter referred to as ‘*KCNV2* KO’) clones by methods described previously [15], and ROs underwent comparative histological analysis at day 150, day 180, and day 280 of differentiation. At day 150, ROs derived from all cell lines possessed rod and cone photoreceptors with outer segments (Rhodopsin, PDE6β, ABCA4, RetGC, L/M Opsin), bipolar cells (PKCa), and Müller glia (CRALBP) in appropriate cell layers, as well as two synaptic layers akin to the outer plexiform and inner plexiform layer (Vglut) (Figure 1D). Expression of *KCNV2* was assessed by QPCR, Wes, and immunohistochemistry (IHC). Both patient and *KCNV2* KO organoids were null for Kv8.2 protein by histology and Wes on whole organoid lysates (Figure 1E). Reduced, but not undetectable, levels of *KCNV2* mRNA were present in *KCNV2* KO and patient-derived ROs (Figure 1F), suggesting a combination of partial nonsense mediated decay at the mRNA level and degradation of truncated proteins resulting from premature stop codons present in both the patient and KO cell lines. There were no signs of degeneration in the *KCNV2* KO ROs at day 180, with mean ONL thickness and cone cell distribution not significantly reduced (Figure 1G,H). Contrary to observations in mouse models, *KCNV2* KO ROs had a small but significant increase in cone cell density. At day 280, average ONL thickness had declined significantly relative to day 150 in both cells lines but there were no significant differences between the two lines at either time point (Figure 1G).

### 2.3. Optimisation of KCNV2 AAV Vectors for Human Photoreceptor Delivery

**Figure 2:**
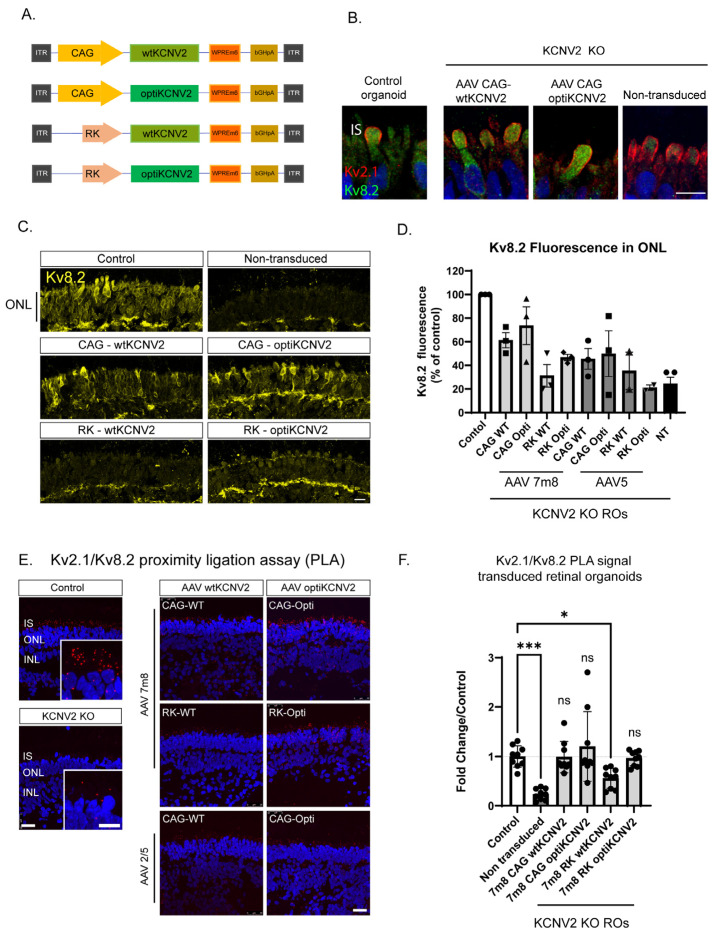
AAV vector-derived Kv8.2 expression, localisation, and interaction with native Kv protein Kv2.1 in transduced ROs. (**A**) Schematic of transgene constructs: AAV2 ITRs, promoter, transgene WPRE6, and bGH poly A. (**B**) High-magnification confocal IHC image of Kv2.1 and Kv8.2 immunolocalisation in the inner segment of control vs. *KCNV2* KO ROs transduced with AAV5-CAG-wt*KCNV2* and AAV5-CAG-opti*KCNV2* vectors. Scale bar = 10 µm. (**C**) Kv8.2 IHC in *KCNV2* KO ROs transduced with 4 vectors packaged in AAV7m8 capsids. Scale bar = 10 µm. (**D**) Quantification of Kv8.2 expression in the ONL of AAV transduced organoids relative to control organoids (*n* = 3 confocal images from *N* = 3 independent differentiations and transductions). (**E**) PLA on organoid cryosections from control, *KCNV2* KO non-transduced, and *KCNV2* KO organoids transduced with AAV7m8 and AAV5 vectors. Red puncta are present where Kv2.1/Kv8.2 heteromers are detected. Scale bar = 25 µm and 10 µm inset. (**F**) Quantification of PLA puncta in the ONL of transduced ROs. Data were normalised to the analysed area and expressed as a fold change relative to control organoids from the same cryoblock and staining session. Each dot represents analysis from a separate organoid (*n* = 3 organoids from *N* = 3 differentiation and transduction experiments). Error bars show the standard deviation. Dotted line shows PLA signal in control organoids. There was no significant difference between control organoids and any of the *KCNV2* KO AAV transduced organoids in PLA puncta per area except AAV7m8-RK-wt*KCNV2*. Kruskal–Wallis test followed by Dunn’s multiple comparison test. Adjusted *p* value *** *p* = 0.0004, * *p* = 0.04, ns = non-significant.

Four *KCNV2* transgene constructs were designed for RO testing (Figure 2A). The wild-type *KCNV2* coding sequence OMIM 607604 (wt*KCNV2*) was used, as well as a codon-optimised version (opti*KCNV2*) designed to enhance translation efficiency by reducing the usage of rare codons and improving G/C dinucleotide distribution (Supplementary Table S10). Both versions were used in combination with the ubiquitously active cytomegalovirus enhancer and chicken β-actin (CAG) promoter and the rhodopsin kinase (RK) promoter which is active in rods and cones. All transgene constructs included a Woodchuck Hepatitis Virus Posttranscriptional Regulatory Element (WPREm6) and a polyA sequence derived from bovine growth hormone (bGH polyA) (Figure 2A). All expression cassette combinations were packaged into AAV5, a naturally occurring AAV serotype with a clinical track record for subretinal delivery to retinal cells [18] and AAV7m8, an engineered AAV2 variant developed for intravitreal gene delivery, which we and others have shown to more efficiently transduce human photoreceptors in RO cultures compared to natural AAV serotypes [15,16,19,20]. In GFP reporter assays at a dose of 5 × 10^10^ VGs per organoid, AAV7m8 has an approximately ten-fold higher transduction efficiency than AAV5 in photoreceptors (mean 40.5 ± 2.2% SD vs. 3.4 ± 0.7% GFP-positive cells; Supplementary Figure S2A). *KCNV2* KO and *KCNV2* patient organoids were transduced at day 130–150 with 3 × 10^11^ viral genomes (VGs) per organoid and harvested three weeks later. Subcellular localisation of AAV5- and AAV7m8-derived Kv8.2 protein was confirmed in organoid cryosections; Kv8.2 protein was detectable in transduced photoreceptor cell bodies and enriched in inner segment structures where it co-localised with Kv2.1 (Figure 2B,C; Supplementary Figure S2B–D). In order to quantify relative vector potency, total Kv8.2 fluorescent signal was measured in the total ONL area of *KCNV2* KO transduced organoids (*n* = 3 organoids from *N* = 3 independent differentiations) and expressed relative to levels in isogenic control organoids of the same developmental age (Figure 2D). At a dose of 3 × 10^11^ VGs, *KCNV2* vectors resulted in Kv8.2 protein levels in the ONL that were higher in organoids transduced with AAV7m8 vectors relative to AAV5 and higher in vectors containing CAG promoters (Figure 2C,D). Quantitative polymerase chain reaction (QPCR) similarly showed increased vector-derived mRNA from AAV7m8 vectors and CAG promoters (Supplementary Figure S2E,F). Codon optimisation delivered an improvement in total protein levels in both CAG and RK promoter containing vectors, which was particularly striking for the AAV7m8 RK promoter vectors, where wt*KCNV2* protein levels were not detectable above background (Figure 2D). The highest expressing vector, AAV7m8-CAG-opti*KCNV2*, had a mean of 74% of wild-type Kv8.2 protein levels in the photoreceptor ONL (Figure 2C,D). Similar results were obtained in *KCNV2* patient samples transduced with AAV7m8 vectors (Supplementary Figure S2E,F).

Ectopically expressed Kv8.2 can only be functional as a heteromeric complex with Kv channels such as Kv2.1 expressed in the photoreceptor IS. The Proximity Ligation Assay^®^ (PLA) allows the visualisation and quantification of in situ, endogenous protein–protein interactions, thus enabling quantification of functional vector-derived Kv8.2 in Kv8.2/Kv2.1 heteromers (Figure 2E). Restoration to control levels of protein–protein interactions was achieved by both CAG vectors and RK-opti*KCNV2*, suggesting these vectors deliver sufficient *KCNV2* protein to occupy Kv2.1 channels to levels seen in the control ROs (Figure 2F), thereby restoring photoreceptor potassium channel homeostasis.

### 2.4. Comprehensive Single-Cell Transcriptomic Analysis Uncovers Cellular Heterogeneity in Retinal Organoids and Stress-Related Pathway Alterations in KCNV2 Disease Model

Single-cell RNA sequencing (scRNA-seq) was employed to further assess the impact of AAV-opti*KCNV2* transduction in *KCNV2* knockout and patient ROs at the transcriptional level. ROs were transduced with AAV7m8 vectors—the higher transducing serotype—at a dose of 1.0 × 10^11^ VGs per organoid in mature organoids (day 200+). Two independent scRNA-seq experiments, *KCNV2* KO scRNA-seq and *KCNV2* patient scRNA-seq, were completed (Supplementary Table S9). The *KCNV2* KO scRNA-seq project comprised non-transduced control (CON-1) and *KCNV2* KO (KO-NT) ROs, and *KCNV2* KO ROs transduced with AAV7m8-RK-opti*KCNV2* (KO-RK) or AAV7m8 CAG-opti*KCNV2* (KO-CAG), and were harvested at day ~280 (control = day 283, *KCNV2* KO = day 279) at week 5 post-transduction and sequenced. In a separate experiment, *KCNV2* patient scRNA-seq, non-transduced control (CON-2 and CON-3) and *KCNV2* patient (PT-NT), alongside *KCNV2* patient organoids transduced with AAV7m8-RK-opti*KCNV2* (PT-RK) or AAV7m8-CAG-opti*KCNV2* (PT-CAG), were harvested at day ~205 of age (control = day 201, *KCNV2* patient = day 205) 5 weeks post-transduction and sequenced. Downstream analyses were performed independently.

**Figure 3:**
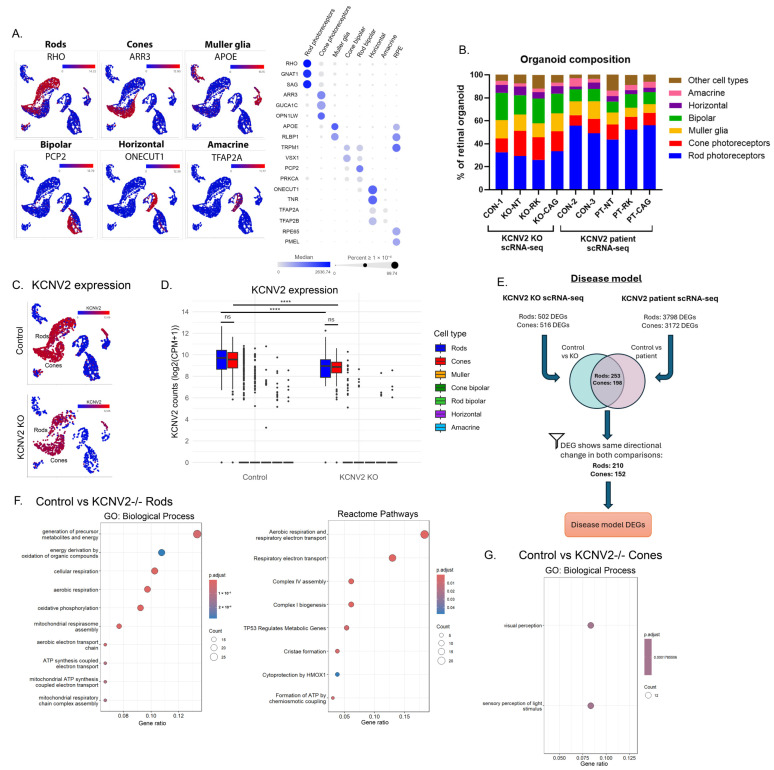
Analysis of retinal organoid composition by single-cell RNA sequencing. (**A**) Uniform manifold approximation and projection for dimension reduction (UMAP) plots and bubble map detailing expression of known biomarkers for the main cell types identified in ROs. UMAP plots shown contain four RO samples control: (CON-1), *KCNV2* KO (KO-NT), and *KCNV2* KO transduced with RK-opti*KCNV2* (KO-RK) or CAG-opti*KCNV2* (KO-CAG). UMAP expression shown in log_2_(CPM + 1). Expression per cell is shown on a gradient from blue (no expression) to red (highest expression). Bubble map colour gradient represents median expression levels of the gene in each cell type cluster of CON-1; bubble size represents the percentage of cells per cluster expressing each gene (expression ≥ 1 × 10^−4^ CPM threshold; CPM = counts per million). (**B**) Quantification of cell type as a percentage of each RO. The cluster ‘Other cell types’ contains the small number of cells identified as RPE cells, transitional cells, astrocytes, fibroblasts, and extracellular matrix cells. Each bar represents *n* = 1 RO. (**C**) UMAPs indicating *KCNV2* expression in control (CON-1) and *KCNV2* KO (KO-NT) RO samples. Expression per cell is shown on a gradient from blue (no expression) to red (highest expression). (**D**) Boxplot quantification of *KCNV2* expression (log_2_(CPM + 1)) per cell type in control (CON-1) and *KCNV2* KO (KO-NT) ROs. Cell types shown on the graph are in the same order as shown in the legend. Kruskal–Wallis test followed by Dunn’s multiple comparison with Benjamini–Hochberg correction. Adjusted *p*-values shown only for rods and cones: control rods vs. control cones ns *p* = 0.362, KO rods vs. KO cones ns *p* = 0.483, control rods vs. KO rods **** *p* = 6.86 × 10^−10^, control cones vs. KO cones **** *p* = 2.80 × 10^−6^. ns = non-significant. (**E**) An overview of the scRNA-seq workflow to establish differentially expressed genes (DEGs) between control and *KCNV2* KO/patient rods and cones. These rod and cone gene sets are defined as ‘Disease model DEGs’. (**F**) Over-representation analysis of disease model DEGs for rod photoreceptors, showing gene ontology biological processes (GO:BP) and reactome pathways that are enriched in the gene set. (**G**) Over-representation analysis of disease model DEGs for cone photoreceptors, showing GO:BP that are enriched in the gene set.

We first sought to characterise the transcriptional profile of control (non-gene edited) vs. *KCNV2*-deficient organoids. With the aid of scType [21], multiple cell type clusters were identified as known retinal cell types (Figure 3A). The ROs in each analysis displayed comparable compositions, which was independent of genotype (Figure 3B). For day 280 ROs evaluated in the *KCNV2* KO scRNA-seq project, rod photoreceptors were the predominant cell type, comprising, on average, 30.7 ± 2.9% (mean ± standard deviation, *n* = 4) of the total cells per organoid, followed by cone photoreceptors at 19.2 ± 4.8%, and Müller glia at 12.9 ± 2.2%. Day 205 ROs of the *KCNV2* patient scRNA-seq project (*n* = 5) had a similar composition, with a slightly higher proportion of rod cells at 52.1 ± 5.0%.

*KCNV2* mRNA was detected primarily in rod and cone photoreceptors in control, *KCNV2* KO, and patient organoids (Figure 3C; Supplementary Figure S3B), albeit at significantly reduced levels in KO and patient photoreceptors relative to control (Figure 3D; Supplementary Figure S3C). *KCNB1* (encoding potassium channel Kv2.1) was expressed in ~60–85% of rods and ~90–95% of cones with significantly lower expression levels in cones relative to rods for control (CON-1,2,3) and KO-NT ROs, but no significant change in PT-NT (Supplementary Figure S4B,C). *KCNB2* (encoding potassium channel Kv2.2), however, was expressed in ~80–90% of cones and ~10–30% of rods, with significantly higher expression levels in cones (Supplementary Figure S4D,E). No significant difference was observed between genotypes aside from slightly reduced, but significant, expression levels of *KCNB1* and *KCNB2* in patient rods and cones, respectively, relative to their age-matched control (Supplementary Figure S4). These data on Kv2 channel distribution across the retina support histological findings in human and macaque tissue [1] and our own histological data (Figure 1A).

Differential gene expression analysis between isogenic control, *KCNV2* KO, and *KCNV2* patient organoids was performed to determine transcriptional changes associated with *KCNV2* deficiency (Supplementary Figure S3E,F; Supplementary Table S1). An overview of the analysis workflow is shown in Figure 3E. Gene lists were filtered to include only those that were differentially expressed (showed significant change in expression levels and a fold change of ±1.2) in both *KCNV2* KO and patient photoreceptors relative to their age-matched controls, and displayed the same directional change (up- or downregulated). In rods, a total of 139 genes were upregulated and 71 genes were downregulated in *KCNV2* KO and patient ROs relative to control, whilst in cone photoreceptors, 79 genes were upregulated and 73 genes were downregulated. Functional analysis of these differentially expressed genes (DEGs) identified an enrichment of gene ontology biological processes (GO:BP) and pathways (Reactome) associated with aerobic respiration (GO:0009060 adj.*p* = 2.79 × 10^−10^) and oxidative phosphorylation (GO:00006119 adj.*p* = 3.66 × 10^−11^), mitochondrial outer membrane permeabilisation (GO:0097345 adj.*p* = 0.0187), *TP53* regulates metabolic genes (R-HSA-5628897 adj.*p* = 0.00534), and cytoprotection by *HMOX1* (R-HAS-9707564 adj.*p* = 0.0481) (Figure 3F; Supplementary Table S2), all of which were confirmed to be dysregulated in *KCNV2* KO rods. Driving the functional enrichment of mitochondrial and stress response processes, multiple members of the gene families NADH dehydrogenase (*NDUF*), cytochrome C oxidase (*COX*), and ATP synthase (*ATP5*) were identified as significantly upregulated in *KCNV2*-deficient rods and encode subunits of mitochondrial Complex I, Complex IV, and Complex V, respectively. Other interesting DEGs of note were multiple members of the solute carrier (*SLC*) family, including the downregulation of the Na^+^/K^+^/Ca^2+^ exchanger *SLC24A1*, which, when dysfunctional, leads to congenital stationary night blindness (CSNB) [22] and upregulation of the potassium channel interacting protein *KCNIP2*, which has been reported to regulate Kv4 (*KCND*) channels [23].

Between control and *KCNV2*-deficient cones, GO analysis indicated an enrichment of DEGs associated with visual perception (GO:0007601 adj.*p* = 1.75 × 10^−4^) and the sensory perception of light stimulus (GO:0050953 adj.*p* = 1.79 × 10^−4^) (Figure 3G; Supplementary Table S2). In common with DEGs in rods, a number of *SLC* family genes were differentially expressed between control and *KCNV2*-deficient cones but, interestingly, in contrast to rods, no genes from the *NDUF*, *COX*, or *ATP5* families were identified. Also differentially expressed relative to control cones were several genes with links to retinal health, including upregulation of *CEBPD* (also DE in rods), which displays increased expression in the degenerating retina of *rd10* mice [24]; downregulation of *NCL*, which regulates the splicing of *NXNL1* (encoding *RdCVF* and *RdCVFL*) and is essential for cone cell survival [25,26]; and downregulation of the vitamin A transporter *STRA6*, mutations in which are associated with the severe ophthalmic abnormalities of Matthew–Wood Syndrome [27,28]. Genes implicated in signal transduction via synaptic connections in the retina were significantly downregulated relative to control cones, including *CAMK2B* [29] and *CPLX3* [30], although there was no significant enrichment in synapse-associated GO terms. Genes involved in the regulation of the stress response and apoptosis were also differentially expressed, although these pathways were not significantly enriched. *RCAN1*, *CRHBP*, *HSPA1A*, and *BCO2* were upregulated and *TRIB3* (also DE in rods) and *DUSP2* were downregulated, correlating with an increase in the response to oxidative stress and apoptosis in *KCNV2*-deficient cones.

### 2.5. Transcriptomic Profiling of AAV-optiKCNV2 Demonstrates RK Promoter Specificity for Rods and Cones Versus Ubiquitous Expression with CAG in Retinal Organoids

**Figure 4:**
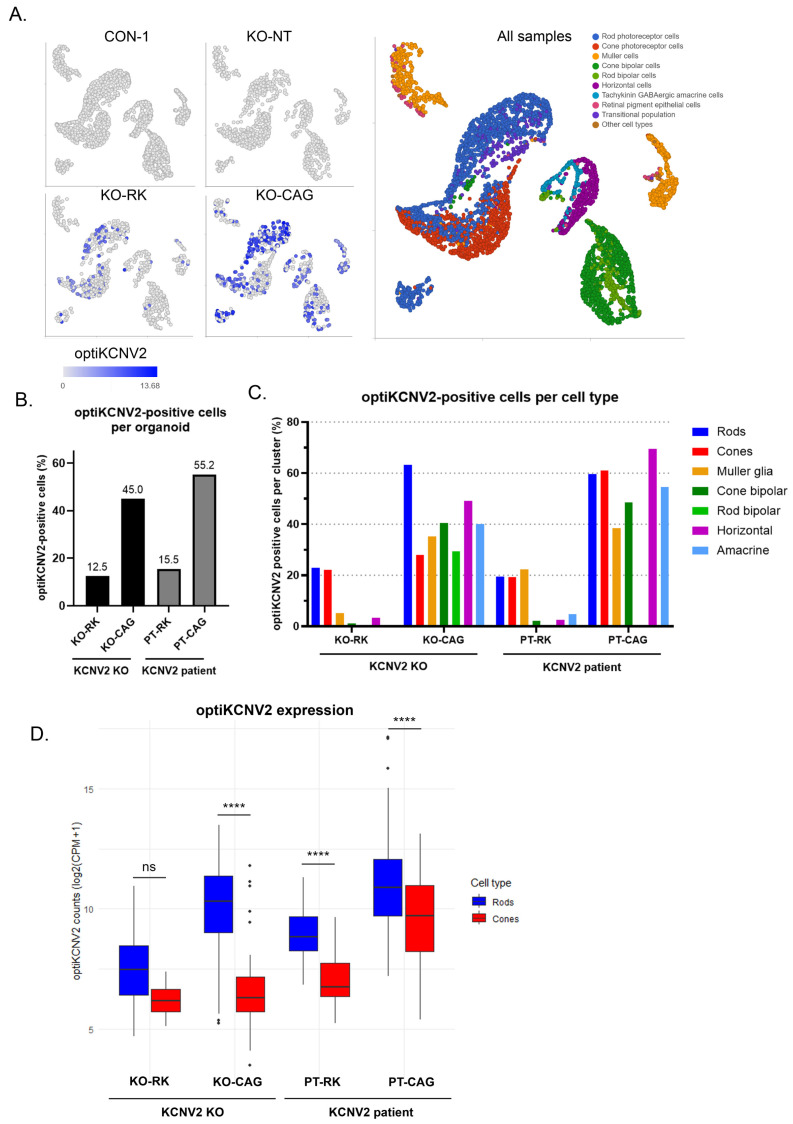
Vector transduction assessment by single-cell RNA sequencing. (**A**) UMAP plots showing opti*KCNV2* expression in control (CON-1) and *KCNV2* KO ROs transduced with AAV7m8-RK-opti*KCNV2* (KO-RK) or AAV7m8-CAG-opti*KCNV2* (KO-CAG) at 1.0 × 10^11^ VG/organoid, or non-transduced (KO-NT). The UMAP plot to the right is coloured by cell type cluster to aid the identification of each cell type. (**B**) Quantification of the percentage of cells per RO expressing opti*KCNV2* in *KCNV2* KO and *KCNV2* patient ROs transduced with opti*KCNV2* vectors at 1.0 × 10^11^ VG/organoid. Each bar represents one RO sample. (**C**) Quantification of the percentage of each cell type expressing opti*KCNV2* in *KCNV2* KO and *KCNV2* patient ROs transduced with opti*KCNV2* vectors at 1.0 × 10^11^ VG/organoid. (**D**) Quantification of opti*KCNV2* expression levels (log_2_(CPM + 1)) in rod and cone photoreceptors positive for opti*KCNV2* mRNA for *KCNV2* KO and patient organoids transduced with RK-opti*KCNV2* (KO-RK and PT-RK) or CAG-opti*KCNV2* (KO-CAG and PT-CAG) vectors. Kruskal–Wallis test followed by Dunn’s multiple comparison with Benjamini–Hochberg correction. Adjusted *p*-values for rods vs. cones: KO-RK ns *p* = 0.0895; KO-CAG **** *p* = 2.69 × 10^−18^; PT-RK **** *p* = 3.89 × 10^−9^; PT-CAG **** *p* = 4.36 × 10^−15^.

scRNA-seq was further employed to evaluate vector performance. AAV7m8 opti*KCNV2* constructs were used, given their higher protein expression levels, and both the RK and CAG promoters were tested. Vector-derived opti*KCNV2* was easily distinguished from endogenous *KCNV2* by virtue of its unique sequence. Quantification of opti*KCNV2* transduction for the whole organoid (Figure 4A,B; Supplementary Figure S5) and main retinal cell types identified (Figure 4C) confirmed that the transduction profile of each vector was comparable between *KCNV2* KO and patient ROs. AAV7m8-RK-opti*KCNV2* transduced 12.5% and 15.5% of total *KCNV2* KO and patient cells respectively, and AAV7m8-CAG-opti*KCNV2* transduced 45.0% and 55.2% of *KCNV2* KO and patient cells, respectively (Figure 4B).

In organoids transduced with opti*KCNV2*-expressing vectors driven by the RK promoter, expression was highest in rods and cones. Rod and cone photoreceptors displayed equivalent numbers of transduced cells, with 21.3 ± 2.3% of rods (mean ± SD of KO-RK and PT-RK, *n* = 2) and 20.8 ± 2.1% of cones expressing the transgene (Figure 4C); however, opti*KCNV2* expression levels per cell (in transduced cells only) was notably higher in rods vs. cones in both cell lines (but only significant in PT-RK), suggesting higher RK promoter activity in rod cells (Figure 4D). Little transduction was observed in the other retinal cell types, with the exception of Müller glia cells, which, interestingly, had a similar percentage of opti*KCNV2*-positive cells to cone cells in the day 205 PT-RK organoid but reduced transduction efficiency in the older, d280 KO-RK organoid. This may reflect differences in the developmental age of the patient and KO organoids (day 205 vs. 280) with younger Müller glia having higher RK promoter activation.

In ROs transduced with opti*KCNV2* driven by a ubiquitous CAG promoter, transgene mRNA was detected in most of the major retinal cell types including horizontal and amacrine cells (Figure 4C). Transduction was most efficient in rods of the older KO-CAG organoid and equal across rods and cones in the younger PT-CAG organoid. Interestingly, opti*KCNV2* expression levels were also significantly higher in rods (Figure 4D), which may reflect a higher promoter activity relative to cones.

### 2.6. Transcriptomic Analysis Reveals AAV-optiKCNV2 Supplementation Partially Rescues Cell Stress Signatures Through Restoration of a Subset of Mitochondrial, Oxidative Stress, and Apoptotic Pathway Genes

**Figure 5:**
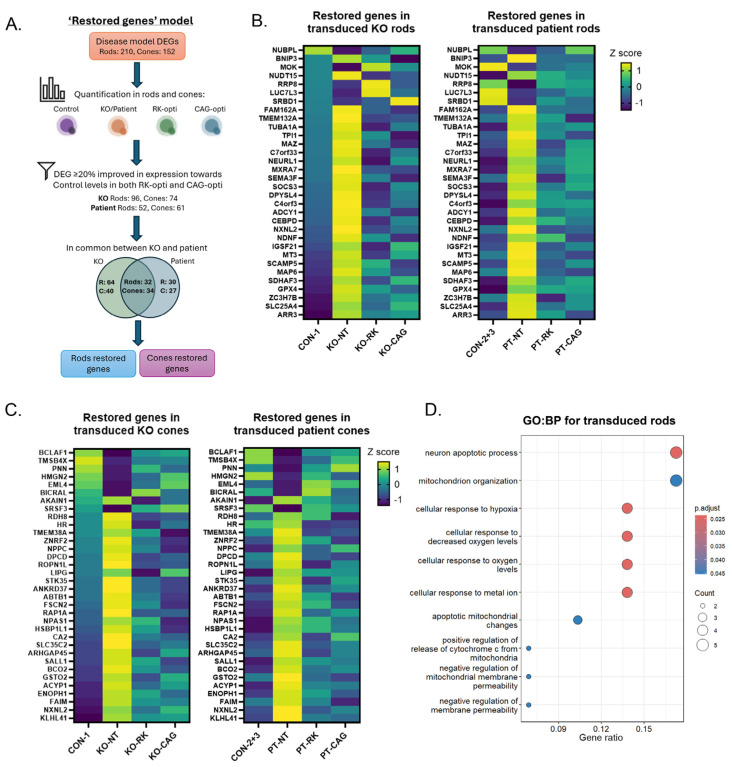
Vector potency assessment by single-cell RNA sequencing. (**A**) Overview of scRNA-seq workflow to determine disease model DEGs (Figure 3E) that are restored upon expression of opti*KCNV2* from either AAV7m8-RK-opti*KCNV2* or AAV7m8-CAG-opti*KCNV2* in *KCNV2* KO and patient rods and cones. (**B**) Heatmaps of restored genes in transduced rod photoreceptors for *KCNV2* KO and patient ROs. Z scores are calculated from the least squares mean (LSMean) of each gene across rod photoreceptors for control (CON-1,2,3), *KCNV2* KO (KO-NT), and *KCNV2* patient samples (PT-NT), and transduced rods for RK-opti*KCNV2* (KO-RK, PT-RK) and CAG-opti*KCNV2* (KO-CAG, PT-CAG) samples. Note: Whilst all comparisons were performed on log_2_(CPM + 1)-normalised counts, LSMean values are computed in CPM by the GSA algorithm in Partek Flow. (**C**) Heatmaps of restored genes in transduced cone photoreceptors for *KCNV2* KO and patient ROs. Z scores are calculated from the LSMean of each gene across cone photoreceptors for control (CON-1,2,3), *KCNV2* KO (KO-NT), and *KCNV2* patient samples (PT-NT), and transduced cones for RK-opti*KCNV2* (KO-RK, PT-RK) and CAG-opti*KCNV2* (KO-CAG, PT-CAG) samples. Note: Whilst all comparisons were performed on log_2_(CPM + 1)-normalised counts, LSMean values are computed in CPM by the GSA algorithm in Partek Flow. (**D**) Dot plot of enriched GO:BP terms for restored genes in rods.

In order to evaluate vector potency, the propensity of AAV-derived opti*KCNV2* transgene expression to restore the transcriptome of *KCNV2*-deficient rod and cone photoreceptors to that of control photoreceptors was evaluated. An overview of the scRNA-seq workflow employed to determine the restoration of disease model DEGs by opti*KCNV2* vectors is shown in Figure 5A. Between *KCNV2* KO and patient rods vs. their respective age-matched controls, 15% (32/210) displayed ≥20% improvement when transduced with any opti*KCNV2* vector whether driven by an RK or CAG promoter (Figure 5B; Supplementary Table S3). An equivalent analysis was performed for cone photoreceptors whereby, of the 152 DEGs between *KCNV2*-deficient cones and their respective controls, 22.4% (34/152) were restored in expression towards control levels by ≥20% in both RK-opti*KCNV2* and CAG-opti*KCNV2* transduced cones (Figure 5C; Supplementary Table S3). DEGs displaying ≥20% improvement in expression levels upon opti*KCNV2* transduction are hereafter referred to as ‘restored genes’.

Functional enrichment analysis of genes restored in rods by replacement of *KCNV2* (Figure 5D; Supplementary Table S4) revealed an over-representation of pathways regulating mitochondrial membrane permeability (GO:0035795; adj.*p* = 0.0452), apoptosis (GO:0051402; adj.*p* = 0.0238), and the response to hypoxia (GO:0071456; adj.*p* = 0.0238; GO:36294 adj.*p* = 0.0238). Interrogation of the gene sets identified for each biological process indicated a reduction in the pro-apoptotic genes *FAM162A*, *BNIP3*, and *SLC25A4* in opti*KCNV2* transduced rods relative to non-transduced *KCNV2* KO rods. These genes are also induced in response to oxidative stress alongside *MT3*, *NDNF*, and *GPX4*, all of which show restoration in expression when *KCNV2* KO or patient-derived rods express the opti*KCNV2* transgene.

Whilst no GO Biological Processes or Reactome pathways were significantly enriched for restored genes in transduced cones, many genes associated with oxidative stress and apoptosis pathways, such as *BCLAF1*, *FAIM*, *SALL1*, *GSTO2*, *ACYP1*, and *SRSF3*, which were dysregulated in *KCNV2*-/- cones, were brought closer to control levels upon expression of opti*KCNV2*. Moreover, restoration of genes which regulate synaptic function and plasticity, *RAP1A* and *NPAS1*, was observed.

Some genes were restored by transduction such that their expression no longer differed significantly from expression in control cones: *DPCD*, which is involved in cilia formation [31,32]; the potassium channel *TMEM38A*; and the visual transduction protein *RDH8* [33,34].

## 3. Discussion

In this manuscript we describe the use of an IPSC retinal organoid disease model for *KCNV2* retinopathy as a tool for development of a novel AAV gene replacement strategy. Disease model retinal organoids enabled three key evaluations: (1) validation of a codon optimisation strategy to improve protein production levels without compromising localisation and native protein–protein interactions, (2) assessment of promoter specificity in a multilayered neural retina, and (3) assessment of the transcriptomic impact of *KCNV2* gene replacement on rod and cone photoreceptors. Importantly, data obtained from this physiologically relevant platform assessing gene therapy vector elements in the context of the human retina enabled us to select a lead clinical candidate vector genome encoding a codon-optimised *KCNV2* transgene from a RK photoreceptor-specific promoter.

*KCNV2* retinopathy is an ideal candidate for classical gene replacement therapy utilising AAV, as it is a monogenic disorder which has relatively slow progression [6], caused by a gene that fits within AAV packaging limits. To translate this therapeutic potential into a clinically viable approach, robust preclinical models are essential. ROs offer such a platform and can significantly reduce the burden of preclinical development that is traditionally placed on animal models. In addition to proof-of-concept work, every aspect of vector design can be evaluated to maximise the chances of clinical success, ensuring that incorporated vector elements (e.g., promoters) are active in the clinically relevant target tissue. The efficiency of AAV capsids in photoreceptor transduction can also be modelled using ROs. Here we show that natural serotypes (AAV2, AAV5, AAV8) used in late-stage clinical programs for subretinal delivery have relatively poor transduction in comparison to engineered AAV variant AAV7m8 in ROs, in agreement with findings from other groups [15,16,19,20].

In this study we utilise RO models both derived from patient blood and using CRISPR/Cas9 to make a *KCNV2* KO. The former has the advantage of possessing the *KCNV2* mutation in the genetic context which is proven to result in a clinical phenotype, and the latter has the advantage of being paired with its isogenic control cell line, which behaves similarly in terms of differentiation capacity and has less genetic variation, a factor that can confound transcriptomic analysis. Our mature RO models have a rod/cone ratio ranging from 5:1 (~day 205) to 2:1 (~day 283), similar to ratios observed within the human macula at 1 mm from the fovea [35]. Mechanistically, evidence from human and non-human primate eyes suggests that Kv2 potassium channels exist as two subpopulations: in their homomeric form, which mediates a high-voltage-activated current; and as heteromers with Kv8.2, which mediate a low voltage-activated current [1]. Since both subpopulations have an important function in mediating light sensitivity and recovery, it may be important not to saturate all Kv channels with Kv8.2. The proximity ligation data presented here gives an indication of the extent to which Kv channels are saturated relative to control organoids; in this system, only AAV7m8-CAG-optiKCNV2 delivered higher-than-wild-type Kv2.1/Kv8.2 heteromers in one out of three separate transduction experiments. This may be an indication that employing a tissue-specific promoter, such as RK, is physiologically more relevant and could ensure channel component homeostasis following intervention, something that might not be the case with the use of a strong constitutive promoter like CAG. We demonstrate here that endogenous *KCNV2* expression is exclusive to photoreceptors, in agreement with previous histological studies in mouse and primate eyes [1]. A further advantage of the RK promoter, therefore, is the restriction of *KCNV2* transgene expression to these target cells, minimising the potential inherent risks of ectopic expression in other retinal cell types.

Beyond our in vitro modelling, preclinical development of *KCNV2* gene replacement therapy benefits from the existence of a well-characterised mouse model with a similar electroretinogram to human *KCNV2* patients—a reduced a-wave and a ‘supernormal’ b-wave [36,37]. The electrophysiological properties of IPSC-derived ROs have been interrogated via patch clamping [38], calcium reporters [39], and multielectrode array [40,41]; however, responses and/or techniques to interrogate them are not currently sufficiently developed to discern disease model-specific electrophysiological phenotypes.

In addition to electrophysiological similarities, the *KCNV2*-/- preclinical mouse model has demonstrated histological changes including a reduction in cone cell numbers (80% of wild-type) and an overall thinning of the ONL [37]. Cone cell loss has also been detected using adaptive optics scanning light ophthalmoscopy in *KCNV2* patients [42]. These phenotypes were not observed in our RO models, with no reduction in ONL thickness or cone cell density between *KCNV2* KO and control organoids. Retinal degeneration in vivo occurs postnatally and may not be apparent in ROs due to their late fetal stage of development [39], as well as the fact that they are cultured mainly in the dark. Despite this, early cues of photoreceptor degeneration and dysfunction were apparent at the transcriptional level. Single-cell RNA sequencing revealed an upregulation in oxidative stress and apoptosis pathways in *KCNV2* patient and *KCNV2* KO rod and cone cells—suggesting an early cell stress phenotype not yet evident in histological assessments.

Increased homomeric Kv2.1 channels seen in our *KCNV2*-deficient ROs would lead to a disruption of potassium homeostasis following light-induced hyperpolarisation of rod and cone cells. The observed early cell stress phenotype may relate to increased energy demands of *KCNV2*-deficient photoreceptors due to a state of excitotoxicity. This is supported by the increased expression of a plethora of mitochondrial complex subunits in *KCNV2*-/- rods and cones, upregulation of oxidative phosphorylation, and, consequently, production of reactive oxygen species, resulting in oxidative stress. Treatment of *KCNV2* KO and patient ROs with opti*KCNV2*-expressing vectors driven by a photoreceptor-specific RK or ubiquitous CAG promoter restored the expression of numerous DEGs relating to the aforementioned pathways, bridging the gap between the transcriptome of control and *KCNV2*-deficient photoreceptors. Overall, *KCNV2* supplementation had an effect on DEGs connected to pathways of phototransduction, mitochondrial function, oxidative stress, hypoxia, and apoptosis. This is an important observation as, when patients with inherited retinal diseases are treated with gene therapy, the goal is to target photoreceptor cells that have not yet undergone degeneration and to intervene at the stage of disease where gene supplementation can halt or delay the underlying cues of degeneration.

In sum, our *KCNV2* RO model supports the use of gene replacement therapy such that, if treated early enough, *KCNV2* restoration could alleviate the cell stress phenotype observed in rods and cones and prevent the photoreceptor loss observed in patients.

## 4. Materials and Methods

### 4.1. Generation of IPSCs and KCNV2 CRISPR KO IPSCs

Control IPSCs and *KCNV2* KO IPSCs were generated from commercially available human male neonatal dermal fibroblasts (Lonza, Basel, Switzerland, CC-22509). *KCNV2* KO IPSCs were produced by simultaneous reprogramming and gene editing using a previously described method [17] with minor alterations. Cas9 ribonucleoproteins (RNPs) were designed to target *KCNV2* Exon 1 using 3 independent guide RNAs (gRNAs), designed and purchased from Synthego (Redwood City, CA, USA). Neonatal dermal fibroblasts were nucleofected with reprogramming plasmids (pEP4EO2SEN2L, pEP4EO2SET2K, pEP4EO2SEM2K [43], and miR 302/367 [17] and with/without Cas9 RNPs on Cell 4D-Nucleofector™ X Kit (Lonza, Basel, Switzerland), program DS-150 for control/KCNV2 KO, respectively. Individual IPSC colonies were isolated to establish clonal cell lines. IPSC lines were genotyped by Sanger sequencing using forward and reverse primers targeting *KCNV2* exon 1 (Supplementary Table S5) to confirm the presence of *KCNV2* null variants. Three IPSC lines (K5, K12, K28) with a 140-base pair (bp) deletion in *KCNV2* exon 1, leading to a predicted premature stop codon at aa324, were selected for RO differentiation.

### 4.2. Generation of KCNV2 Patient IPSC

PBMCs were collected from a male patient homozygous for nonsense variant *KCNV2*: c778A > Tp.(Lys260*) at Moorfields Eye Hospital. PBMCs were isolated by Ficoll density gradient and expanded in Stemspan 3000 Expansion Medium (STEMCELL Technologies, Vancouver, BC, Canada). On day 6, cells were transfected with reprogramming plasmids in Lonza P3 Nucleofection solution in a Cell 4D-Nucleofector™ X Kit (Lonza), according to manufacturer instruction (20 µL/electroporation reaction).

### 4.3. Generation of Retinal Organoids and AAV Transduction

*KCNV2* KO IPSCs, *KCNV2* patient, and control IPSCs were maintained in E8 Flex (A28583-01, Thermo Fisher Scientific, Waltham, MA, USA) on rh-Laminin 521 (A29249, Gibco, Mölndal, Sweden), passaged twice per week at a 1-in-12 split ratio using Versene (15040-066, Thermo Fisher Scientific, Oxford, UK). They were differentiated towards a 3D RO fate following previously published protocols [8,44]. Briefly, IPSCs were grown in 6 well plates (3598, Corning Costar, New York, NY, USA) to 90–100% confluence in E8 Flex media before being switched to E6 media (A15165-01, Thermo Fisher Scientific, UK) for two days. Neuroretinal fate induction occurred in PIM media (Advanced DMEM (12491-015), Thermo Fisher Scientific, UK), 1% N2 (17502-048, Thermo Fisher Scientific, USA), 1% Glutamine (25030-081, Thermo Fisher Scientific, UK), 1% NEAA (11140-050, Thermo Fisher Scientific, UK). Between day 25 and 35 post-seeding, neuroretinal vesicles were manually dissected and grown in suspension (96 well plates, 174929 Nunclon Sphera, Thermo Fisher Scientific, Tokyo, Japan). At day 35, retinal differentiation media (RDM, Advanced DMEM, 1% B27 minus vitamin A (A33535-01, Thermo Fisher Scientific, USA) was used until day 50, and RDM + F (DMEM/F12(31331-028), Thermo Fisher Scientific, UK), 2% B27 minus Vitamin A, 10% FBS (A56708-01, Gibco, Grand Island, NY, USA), 2% Glutamax (35050-038, Thermo Fisher Scientific, UK), 100 µm Taurine (T4571, Sigma-Aldrich, Tokyo, Japan) until day 70; during this time, media was exchanged three times per week. At day 70, organoid quality was assessed for the presence of transparent neuroepithelium and transferred to 24-well low attachment plates (3473, Corning Costar, New York, NY, USA), in which media was exchanged twice per week. ROs were cultured in Alt70 media (Advanced DMEM, 2% B27—vitamin A, 10% FBS, 1% Glutamax, Taurine 100 µm, and 1 µm retinoic acid (207341000, Fisher Scientific, Shanghai, China). At day 90, the media was changed to Alt90 media (Advanced DMEM, 2% B27 without vitamin A, 1% N2, lipid mixture 1:1000 (Sigma L5146, Sigma, Livonia, MI, USA), 100 µm Taurine, 2% Glutamax, 7.5 mM Glucose (A16828.36, Thermo Fisher Scientific, Zuiderweg, The Netherlands), and 0.5 µm retinoic acid. At day 120, retinoic acid was no longer added to Alt90 medium. For IHC, PLA, QPCR and Western blots, individual *KCNV2* KO ROs were dosed with 3 × 10^11^ viral genomes (VGs) at day 130–150 of differentiation with AAV-*KCNV2* in a volume of 100 µL, then harvested for analysis 3 weeks post-transduction. For scRNA-seq, *KCNV2* KO and patient organoids were transduced with 1.0 × 10^11^ VG per organoid and harvested 5 weeks post-transduction. A summary of the cell lines used in each Figure in this manuscript can be found in Supplementary Table S8.

### 4.4. Flow Cytometry

Retinal Organoids were dissociated into single cells using the Neurosphere Dissociation Kit (130-123-095-943, Miltenyi Biotec, Bergisch Gladbach, Germany) and stained with photoreceptor specific marker CD73 conjugated to APC (Clone AD2, 130-123-802, Miltenyi Biotec, Germany). The dissociated retinal organoids were washed once with PBS (14190-144, Gibco, London, UK) and incubated with CD73-APC in 0.5% BSA/PBS (BSA, A3059, Sigma Aldrich, Dorset, UK), for 30 min at 4 °C in the dark. After the incubation, the cells were washed twice with ice-cold PBS and resuspended in 350 µL of cold 3% BSA/PBS solution. The resuspended cells were transferred to 5 mL round-bottom polystyrene FACS tubes with 35 µm strainer caps (352235, BD Biosciences, Mexico City, Mexico). Samples were kept on ice till FACS analysis on the BDFACSLyric flow cytometer (BD Biosciences, Shanghai, China).

### 4.5. Codon Optimisation

Codon optimisation was performed by GeneArt (Thermo Fisher). GC-rich regions were removed and the rare codons were replaced with more frequently used synonymous codons.

### 4.6. AAV Production

AAV7m8 and AAV5 RK-wt*KCNV2*, RK-opti*KCNV2*, CAG-wt*KCNV2*, CAG-opti*KCNV2* were produced by Signagen. Recombinant AAV was produced using a helper virus-free triple-transfection method. HEK293T cells were transfected with pAAV-(transgene), pRep2/Cap7m8 (or Cap5), and pHelper plasmids at a ratio of 5:5:8. Seventy-two hours post-transfection, AAV particles were purified from the clarified cell lysate by AAVX affinity chromatography. AAV-containing fractions were subsequently concentrated and formulated using a 100 kDa MWCO protein concentrator in DPBS supplemented with 0.001% Pluronic F-68. Genomic titres (VG/mL) were determined by QPCR (StepOne Plus, Applied Biosystems, Singapore) (Supplementary Table S11). All vectors were analysed together with primer/probes targeting the ITRs. F: GGAACCCCTAGTGATGGAGTT; R: CGGCCTCAGTGAGCGA; Probe: CACTCCCTCTCTGCGCGCTCG. Standards (rAAV reference—American Type Culture Collection) and validation samples (both viral and plasmid) were included in each assay.

AAV2,5,8 and 7m8 CAG-eGFP were produced in-house. Recombinant AAV was generated by helper virus-free triple transfection. HEK293 suspension cells were transfected with pAAV-CAG-eGFP, pRep2/Cap7m8/5/2 or 8, and pHelper at a ratio of 1:1:2. Seventy-two hours after transfection, AAV particles were purified from the clarified lysate by AAVX Affinity chromatography. AAV-containing fractions were concentrated and formulated using a 100 kDa MWCO protein concentrator using a DPBS buffer supplemented with Pluronic F68 (0.001%). Genomic titres (VG/mL) were determined by QPCR (with primers targeting bGH polyA and ultramer standards.

### 4.7. Proximity Ligation Assay and Quantification

Control, *KCNV2* KO non-transduced, and *KCNV2* KO transduced organoids were embedded in the same OCT block and sectioned into 7 µm cryosections. Cryosections were co-stained with Kv8.2 (rabbit 1:400) and Kv2.1 (mouse 1:400) (Supplementary Table S7) antibodies and rabbit and mouse PLA plus and minus probes. Following ligation and amplification steps (duo link—orange), PLA puncta in the outer nuclear layer were visualised at 63× magnification. Confocal images were acquired using a Leica SP8 confocal microscope (Leica, Wetzlar, Germany). Maximum intensity projections were created from Z stacks and utilised for downstream analysis. Single channel images were thresholded in ImageJ (Version 1.53q), and the number of puncta were counted using the ‘analyse particles’ function. The numbers of puncta were then normalised to the total ONL area per image. Data were expressed as a fold change over normalised puncta counts for the control samples acquired from the same block, staining, and imaging session.

For each group, *n* = 3 images were acquired from *n* = 3 sections from *N* = 3 IPSC-organoid differentiations. Significance was determined by Kruskal–Wallis with a post hoc Dunn’s test for multiple comparisons.

### 4.8. Quantification of Immunofluorescence—ImageJ (ONL Thickness, Cone Cell Count, Kv8.2 Fluorescence)

Control, *KCNV2* KO non-transduced, and *KCNV2* KO transduced organoids were embedded in the same OCT block and sectioned into 7 µm cryosections. Sections were stained with Kv8.2 and DAPI (ONL thickness and Kv8.2 fluorescence) or with rhodopsin and L/M opsin (cone cell density). Merged tile scan images of whole organoids from each condition were acquired and exported to ImageJ (Version 1.53q). In the DAPI channel, total ONL area was measured using the polygon selection tool, and ONL length using the segmented line tool. These measurements were used to calculate mean ONL thickness. For Kv8.2 fluorescence intensity, the region of interest (ROI) of the ONL area from the DAPI image was used to measure integrated signal intensity in the Kv8.2 channel, which was then normalised to total ONL area. Finally, the fluorescence intensity was expressed as a percentage of the non-transduced control organoid (‘Control’ in Figure 2D) from the same differentiation, stained, and imaged in the same session. For cone cell density measurements, sections were stained with L/M opsin, rhodopsin, and DAPI. Tile scans at 40× were acquired covering the entire organoid. L/M opsin-positive cones were counted manually over the length of rhodopsin-positive ONL measured with the segmented line tool. Data were then normalised to ONL length.

### 4.9. Protein Extraction and Immunoblotting

Organoids were lysed in radioimmunoprecipitation assay (RIPA; 89900, Thermo Fisher Scientific, USA) buffer containing 1% protease and phosphatase inhibitor cocktail followed by sonication (Model 120 Sonic Dismembrator, Fisherbrand, Waltham, MA, USA). Protein quantification was performed using a Pierce bicinchoninic assay kit (23227, Thermo Fisher Scientific, USA), and 1.5 µg protein lysate was loaded per lane following heating at 70 °C for 10 min. Automated capillary electrophoresis was performed using a Wes instrument by ProteinSimple Instruments (004-600, Biotechne, Minneapolis, MN, USA) and analysed using Compass for SimpleWestern software version 6.3. Primary antibody information can be found in Supplementary Table S7. Compatible secondary antibodies targeting mouse or rabbit were sourced from Biotechne.

### 4.10. RT-qPCR

Total RNA was extracted from ROs using the PicoPure RNA Isolation Kit (Arcturus, San Diego, CA, USA). First-strand cDNA synthesis was performed using the Superscript IV Vilo Master Mix (Thermo Fisher Scientific) using 100 ng of total RNA per sample.

QPCR analysis was performed using TaqMan Fast Advanced Master Mix (Thermo Fisher Scientific) following standard cycling parameters using TaqMan assays to identify genes of interest (Supplementary Table S6). Primers and probes targeting opti*KCNV2* were designed using the IDT PrimerQuest Tool (http://eu.idtdna.com/PrimerQuest/Home; 2 February 2022). The mRNA levels for target genes were normalised to the geometric mean of endogenous reference genes *hGAPDH* and *β-ACTIN* (Supplementary Table S6) and expressed as relative expression versus day 70 (Figure 1) or expression in AAV5-RK-*KCNV2* transduced samples (Supplementary Figure S2).

### 4.11. Histology

ROs were washed in DPBS, fixed in paraformaldehyde fixative solution (Thermo Fisher Scientific, Waltham, MA, USA), washed once with DPBS, and placed in 30% sucrose solution overnight at 4 °C. The following day, ROs were embedded in optimal cutting temperature (OCT) compound before freezing at −80 °C. Organoids were sectioned at 7 µm and stored at −20 °C. Where transduced organoids were being analysed, multiple transduced organoids were mounted on the same block alongside non-transduced and control organoids to minimise variability in staining and image acquisition.

RO sections were stained with primary antibodies (Supplementary Table S7) overnight at 4 °C, before staining with Alexa Fluor 488 or 555 secondary antibodies (Thermo Fisher Scientific). Cell nuclei were identified with 4′,6-diamidino-2-phenylindole (DAPI, Sigma, St. Louis, MO, USA).

### 4.12. Retinal Organoid Dissociation and scRNA-Seq Library Preparation

ROs were shipped live to the Genome Centre at Queen Mary University London (QMUL) where they were dissociated using a Neurosphere Dissociation kit (Miltenyi Biotec), achieving ≥80% viability and ≥1000 cells/µL. The 10× Chromium Single Cell 3′ v3.1 kit was used for the *KCNV2* KO scRNA-seq project, and the 10× Chromium Single Cell 5′ v2 kit for the *KCNV2* patient scRNA-seq project. Samples were then loaded onto the 10× Chromium instrument and the following steps, including single cell droplet generation, followed manufacturers guidelines. All sequencing was paired-end 90 bp format performed using Illumina NextSeq. Cell Ranger (v5.0 for both projects), utilising human genome reference GRCh38 with the addition of a custom reference genome for codon-optimised *KCNV2*, was used for alignment of raw sequencing reads.

### 4.13. Computational Analysis of scRNA-Seq Data

Initial data QC, normalisation, principal component analysis, UMAP generation, and differential expression analyses were performed using Partek Flow software v10.0.21.0718 or later. Cells with <500 UMIs, >20% mitochondrial percentage, and >20% ribosomal percentage were excluded from analysis. Gene expression values were normalised using log_2_-transformed counts per million (CPM) + 1 (log_2_(CPM + 1)) transformation. Cell types were defined with the aid of scType [21] and manual annotation based on published biomarkers of known retinal cell types. Principal component analysis was performed using the top 100 features, followed by UMAP generation with the following parameters: principal components = 7, distance metric = Euclidean, local neighbourhood size = 30, minimal distance = 30, number of iterations = 0. Gene Set Analysis (GSA) was performed using Partek Flow to compare differentially expressed genes between the clusters and conditions specified. Functional enrichment analysis was performed in R v4.4.3 [45] using the relevant packages clusterProfiler v4.14.6 [46], enrichplot v1.26.6 [47] and ggplot2 v3.5.2 [48]. Statistical tests for gene quantification were also run in R, using a Kruskal–Wallis test followed by post hoc Dunn’s analysis, with Benjamini–Hochberg correction for multiple comparisons.

Each scRNA-seq sample is a single RO. Number of cells analysed (after application of QC filters)—*KCNV2* KO scRNA-seq: CON-1 = 2509, KO-NT = 1033, KO-RK = 920, KO-CAG = 853. *KCNV2* patient scRNA-seq: CON-2 = 3804, CON-3 = 3056, PT-NT = 2294, PT-RK = 3088, PT-CAG = 2700.

## 5. Patents

Pending patent application related to expression constructs, viral genomes, and vectors for the expression of Kv8.2 (US20240307559A1).

## Data Availability

Data is contained within the article and Supplementary Materials.

## Supplementary Materials

The following supporting information can be downloaded at: https://www.mdpi.com/article/10.3390/ijms27010449/s1.

## Abbreviations

The following abbreviations are used in this manuscript:

AAV	Adeno-associated virus
bGH	Bovine growth hormone
CAG	CMV enhancer/chicken beta-actin
CDSRR	Cone dystrophy supernormal rod response
CSNB	Congenital stationary night blindness
DE	Differentially expressed
DEG	Differentially expressed gene
GO	Gene ontology
GO:BP	Gene ontology biological processes
IHC	Immunohistochemistry
INL	Inner nuclear layer
IPSC	Induced pluripotent stem cell
IS	Inner segment
KB	kilobase
*KCNV2*	Potassium voltage-gated channel modifier subfamily V member 2
kDa	kiloDaltons
KO	Knockout
ns	Non-significant
ONL	Outer nuclear layer
optiKCNV2	Codon-optimised KCNV2
OS	Outer segment
PBMC	Peripheral blood monocyte cell
PLA	Proximity ligation assay
PT	Patient
QPCR	Quantitative polymerase chain reaction
RK	Rhodopsin kinase
RO	Retinal organoid
scRNA-seq	Single-cell RNA sequencing
VGs	Viral genomes
WPREm6	Woodchuck hepatitis virus posttranscriptional regulatory element
