# Supplementary material for: KCNV2-Deficient Retinal Organoid Model of Cone Dystrophy—In Vitro Screening for AAV Gene Replacement Therapy
Source: Int J Mol Sci. 2025 Dec 31;27(1):449. doi: 10.3390/ijms27010449 (PMC12786086; doi:10.3390/ijms27010449)
Supplement: Supplementary file 1 [file ijms-27-00449-s001.zip › ijms-4014727-supplementary.pdf]

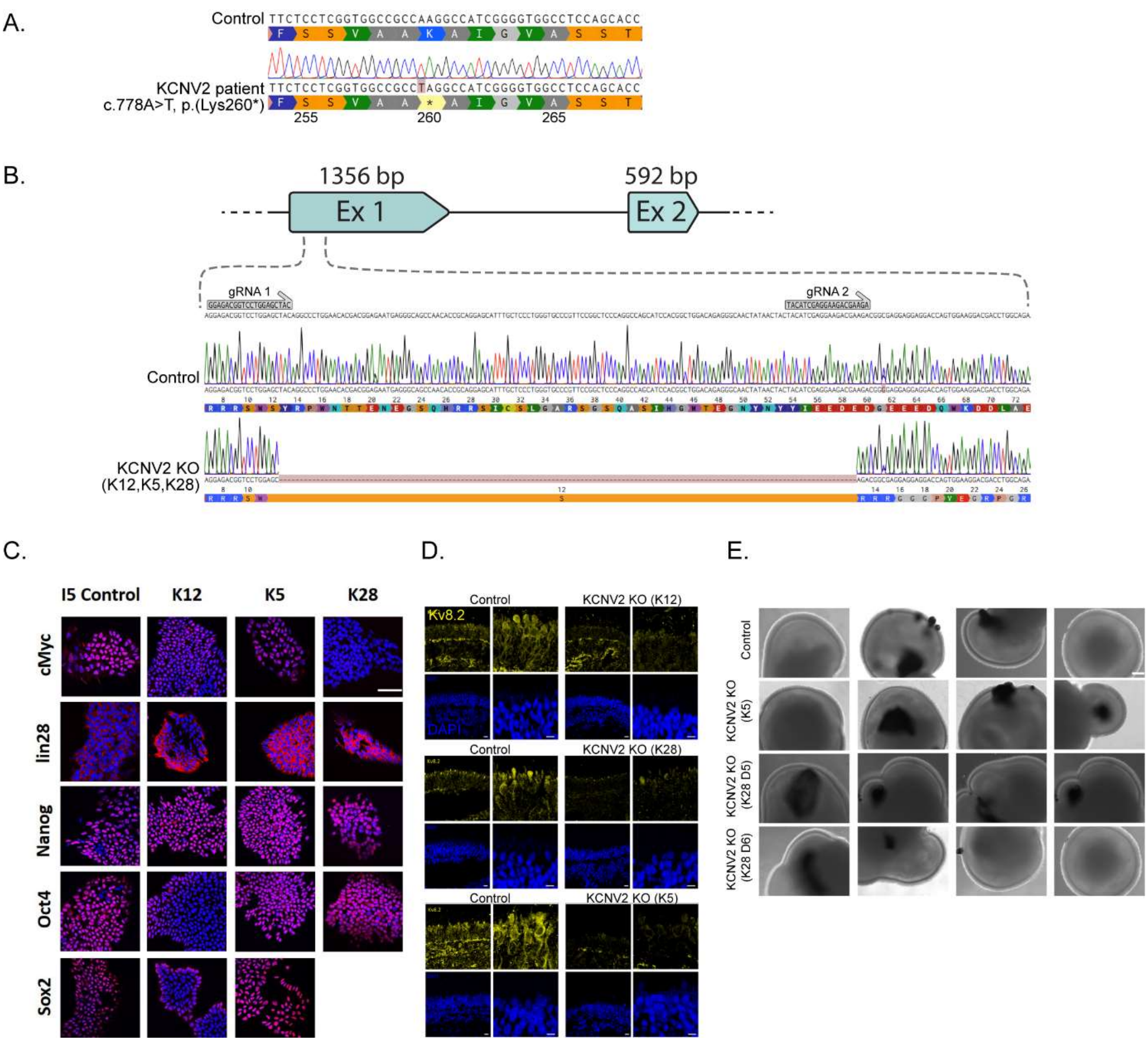

Supplementary Figure S1. Characterisation of *KCNV2* KO and patient IPSC and retinal organoids

A. Sequence traces from *KCNV2* patient blood derived IPSCs homozygous for nonsense variant *KCNV2*: c778A>Tp.(Lys260*) aligned to control IPSCs.

B.Sequence traces from *KCNV2* KO IPSC aligned to non-edited WT sequence. Three clonal lines were derived each with a 140bp deletion in exon 1 leading a frame shift and predicted stop codon at aa 324.

C. Pluripotency staining for control cell line I5 and isogenic KCNV2 KO clones K12, K5 and K28. Scale bar = 50μm

D. IHC for Kv8.2/DAPI in retinal organoids derived from control and KCNV2 KO clones K12,K5 and K28. Scale bar = 10μm.

E. Live brightfield images of representative retinal organoids derived from control and *KCNV2* KO clonal lines showing OS border. Scale bar = 100μm.

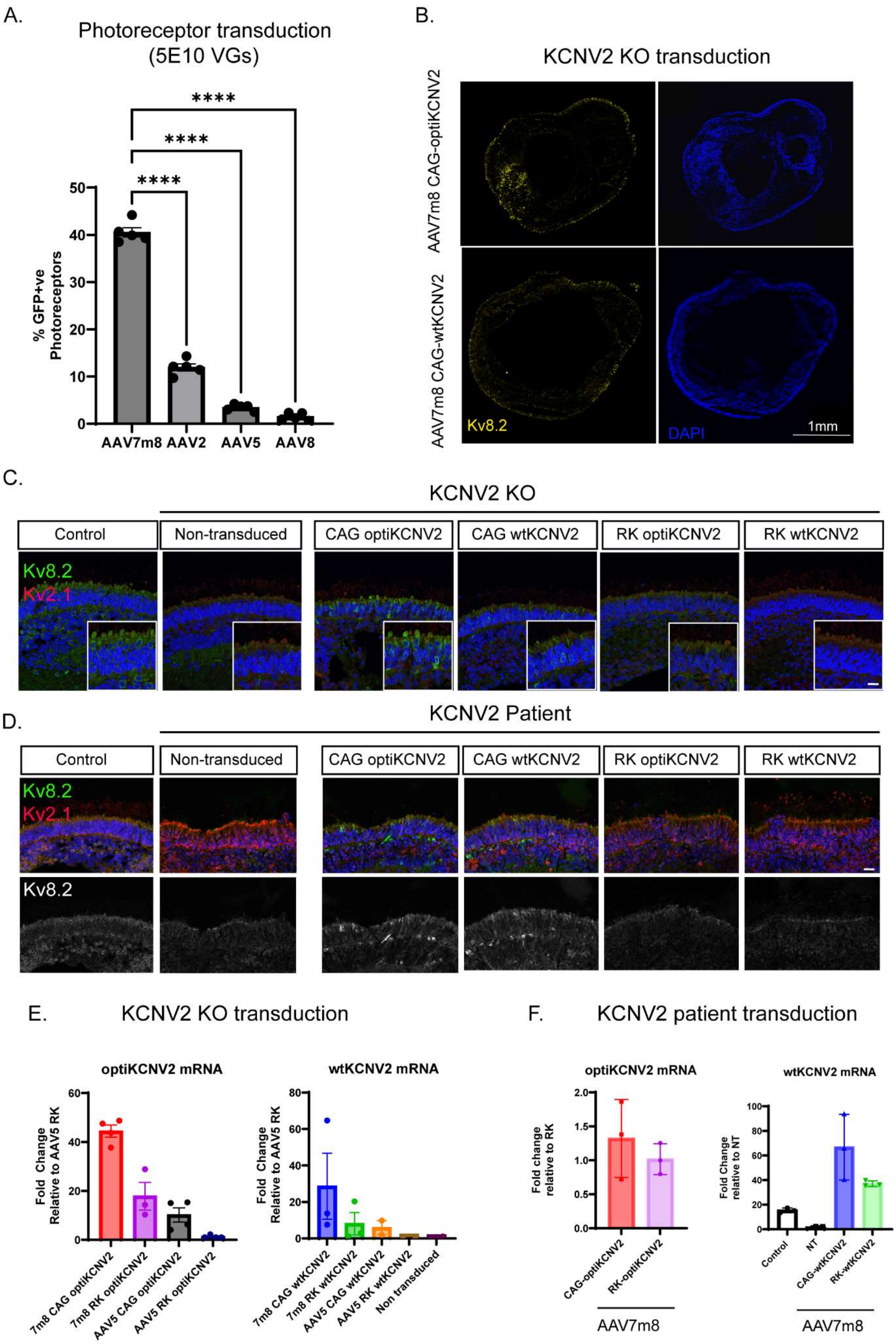
A.
Photoreceptor transduction
(5E10 VGs)
****
****
****
% GFP+ve Photoreceptors
50
40
30
20
10
0
AAV7m8
AAV2
AAV5
AAV8
B.
KCNV2 KO transduction
AAV7m8 CAG-optiKCNV2
AAV7m8 CAG-wtKCNV2
Kv8.2
DAPI
1mm
C.
KCNV2 KO
Control
Non-transduced
CAG optiKCNV2
CAG wtKCNV2
RK optiKCNV2
RK wtKCNV2
Kv8.2
Kv2.1
D.
KCNV2 Patient
Control
Non-transduced
CAG optiKCNV2
CAG wtKCNV2
RK optiKCNV2
RK wtKCNV2
Kv8.2
Kv2.1
Kv8.2
E.
KCNV2 KO transduction
optiKCNV2 mRNA
Fold Change Relative to AAV5 RK
60
40
20
0
7m8 CAG optiKCNV2
7m8 RK optiKCNV2
AAV5 CAG optiKCNV2
AAV5 RK optiKCNV2
wtKCNV2 mRNA
Fold Change Relative to AAV5 RK
80
60
40
20
0
7m8 CAG wtKCNV2
7m8 RK wtKCNV2
AAV5 CAG wtKCNV2
AAV5 RK wtKCNV2
Non transduced
F.
KCNV2 patient transduction
optiKCNV2 mRNA
Fold change relative to RK
2.0
1.5
1.0
0.5
0.0
CAG-optiKCNV2
RK-optiKCNV2
AAV7m8
wtKCNV2 mRNA
Fold Change relative to NT
100
80
60
40
20
0
Control
NT
CAG-wtKCNV2
RK-wtKCNV2
AAV7m8

Supplementary Figure S2. Vector-derived KCNV2/Kv8.2 expression in retinal organoids

A. Flow cytometry data showing transduction efficiency of various capsids in retinal organoids. A CAG-GFP transgene was packaged into the indicated capsids which were dosed at 5E10VGs per organoid (*n*=5 per capsid), incubated for three weeks before dissociation and staining with photoreceptor marker CD73. Bars show GFP positive cells as a percentage of photoreceptors (CD73 positive cells). ****$p<0.0001$. One way ANOVA, post hoc Tukey's multiple comparison test.
B. Low magnification images of AAV7m8-CAG-wtKCNV2 and opti*KCNV2* transduced organoids stained with Kv8.2 antibody showing even distribution of transduction. Scale bar = 1mm.
C. Representative IHC images of *KCNV2* KO organoids transduced with AAV7m8 vectors stained with anti-Kv8.2 (green) and anti-Kv2.1 (red) antibodies. Scale bar = 20µm
D. Representative IHC images of *KCNV2* patient organoids transduced with AAV7m8 vectors stained with anti-Kv8.2 (green and white below) and anti-Kv2.1 (red) antibodies. Scale bar = 20µm.
E. QPCR for opti*KCNV2* and wt*KCNV2* in organoids transduced with AAV5 and 7m8 vectors. Data are as a fold change relative to the lowest expressing AAV5 RK vector.
F. QPCR for wt*KCNV2* mRNA in control and *KCNV2* patient organoids transduced with AAV7m8 vectors expressed relative to non-transduced *KCNV2* patient organoids. opti*KCNV2* mRNA expressed relative to RK-opti*KCNV2* transduced organoids.

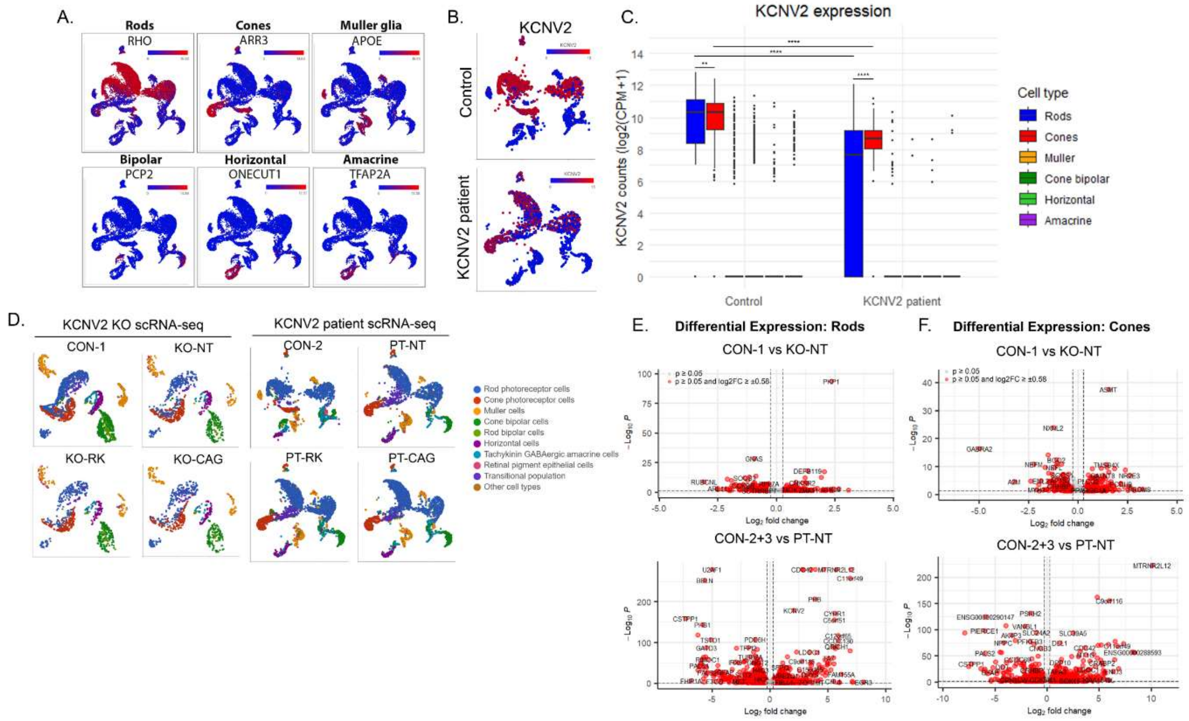

Supplementary Figure S3. Analysis of retinal organoid composition by single cell RNA sequencing

A. UMAP plots of Control (CON-2 and CON-3) and *KCNV2* patient (PT-NT, PT-RK, PT-CAG) ROs combined detailing expression (log_2CPM) of known biomarkers for the main cell types identified in our ROs. UMAP plots shown contain four RO samples Control, *KCNV2* patient, and *KCNV2* patient ROs transduced with RK-optiKCNV2 (RK-opti), and CAG-optiKCNV2 (CAG-opti).
B. UMAPs indicating *KCNV2* expression in control (CON-2 and CON-3) and *KCNV2* patient (PT-NT) RO samples. Expression per cell is shown on a gradient from blue (no expression) to red (highest expression).
C. Boxplot quantification of KCNV2 expression (log_2(CPM+1)) per cell type in control (CON-2 and CON-3) and KCNV2 patient (PT-NT) ROs. Kruskal-Wallis test followed by Dunn's multiple comparison with Benjamini-Hochberg correction. Adjusted p-values shown only for rods and cones: Control rods vs Control cones ***p*=1.21E-3, patient rods vs patient cones *****p*=5.04E-5, Control rods vs KO rods *****p*=2.87E-74, Control cones vs KO cones *****p*=2.87E-12.
D. UMAP plots for each organoid sample in the KCNV2 KO scRNA-seq and KCNV2 patient scRNA-seq projects, coloured by cell type. Cell types were defined with the aid of scType, an automated cell type annotation tool.
E. Volcano plots of differentially expressed genes of control (CON-1) vs KCNV2 KO (KO-NT) rods and control (CON-2 and CON-3) vs patient (PT-NT) rods. Red dots denote genes that have a *p* value ≥ 0.05 and log_2 fold change (log_2FC) ≥ 0.2630 (equivalent to 1.2-fold change between conditions), grey dots represent genes which have a *p* value ≥ 0.05 but do not meet the log_2FC threshold.

F. Volcano plots of differentially expressed genes of control (CON-1) vs KCNV2 KO (KO-NT) cones and control (CON-2 and CON-3) vs patient (PT-NT) cones. Red dots denote genes that have a *p* value ≥ 0.05 and log_2FC ≥ 0.2630 (equivalent to 1.2-fold change between conditions), grey dots represent genes which have a *p* value ≥ 0.05 but do not meet the log_2FC threshold.

Supplementary Figure S4. Transcriptomic analysis of *KCNB1* and *KCNB2* expression in retinal organoids.

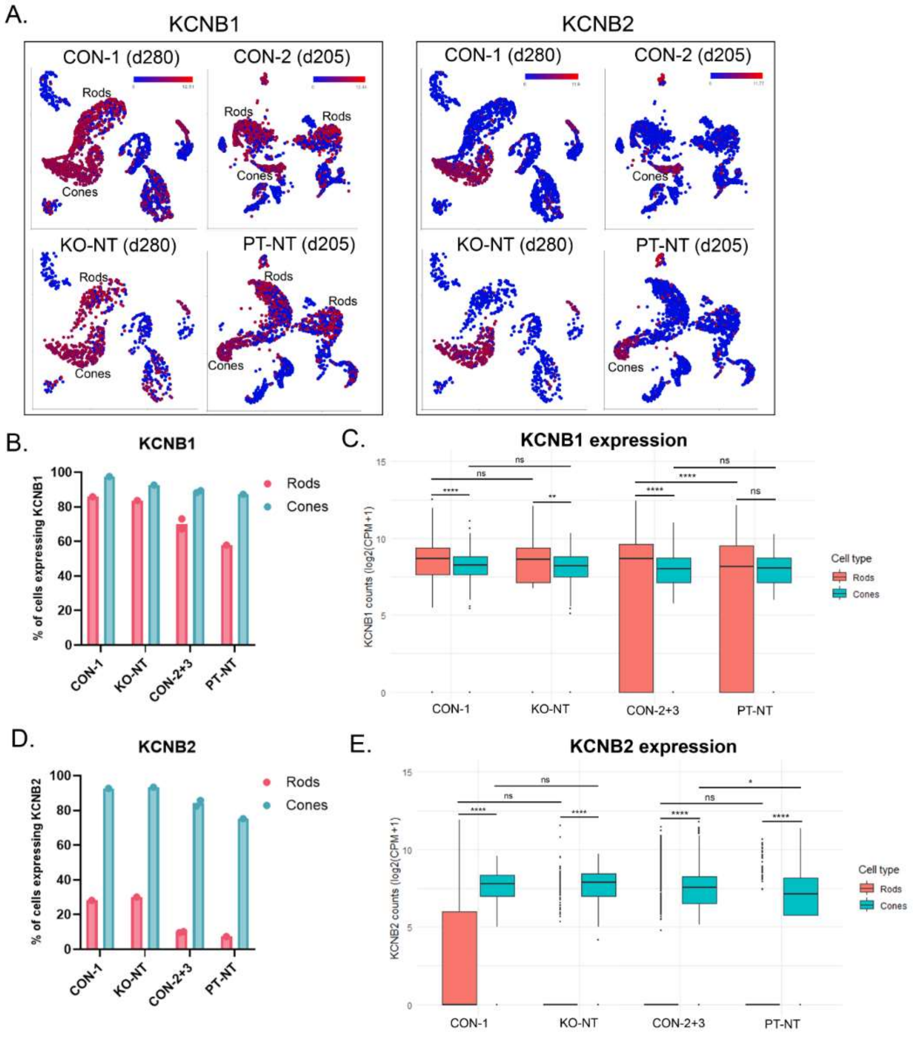

A. UMAP plots of *KCNB1* and *KCNB2* expression in Control (CON-1 and CON-2), *KCNV2* KO (KO-NT), and *KCNV2* patient (PT-NT) ROs. Expression shown in log_2CPM. Red = highest expression, blue = lowest expression.
B. Quantification of the percentage of Control (CON-1) and *KCNV2* KO (KO-NT) rods and cones expressing *KCNB1*.
C. Boxplot quantification of *KCNB1* expression levels (log_2(CPM+1)) in Control (CON-1,2,3), *KCNV2* KO (KO-NT), and KCNV2 patient (PT-NT) rods and cones. Kruskal-Wallis test followed by Dunn's multiple comparison with Benjamini-Hochberg correction. Adjusted *p* values: *KCNB1*: CON-1 rods vs CON-1 cones *****p*=6.51E-5, KO-NT rods vs KO-NT cones ***p*=0.00163, CON-1 rods vs KO-NT rods non-significant (ns) *p*=0.711, CON-1 cones vs KO-NT

cones ns *p*=0.643. CON-2+3 rods vs CON-2+3 cones ****p=7.51E-14, PT-NT rods vs PT-NT cones ns *p*=0.247, CON-2+3 rods vs PT-NT rods ****p=8.49E-6, CON-2+3 cones vs PT-NT cones ns *p*=0.834.

D. Quantification of the percentage of Control (CON-1) and *KCNV2* KO (KO-NT) rods and cones expressing *KCNB2*.

E. Boxplot quantification of *KCNB2* expression levels (log_2(CPM+1)) in Control (CON-1,2,3), *KCNV2* KO (KO-NT), and KCNV2 patient (PT-NT) rods and cones. Kruskal-Wallis test followed by Dunn's multiple comparison with Benjamini-Hochberg correction. Adjusted *p* values: *KCNB2*: CON-1 rods vs CON-1 cones ****p=1.14E-92, KO-NT rods vs KO-NT cones ****p=1.61E-60, CON-1 rods vs KO-NT rods ns *p*=0.608, CON-1 cones vs KO cones ns *p*=1.00. CON-2+3 rods vs CON-2+3 cones **** p=0.0000, PT-NT rods vs PT-NT cones ****p=4.29E-63, CON-2+3 rods vs PT-NT rods ns *p*=0.314, CON-2+3 cones vs PT-NT cones *p=0.0149.

Supplementary Figure S5 – Transcriptomic analysis of optiKCNV2 expression in KCNV2 patient scRNA-seq project

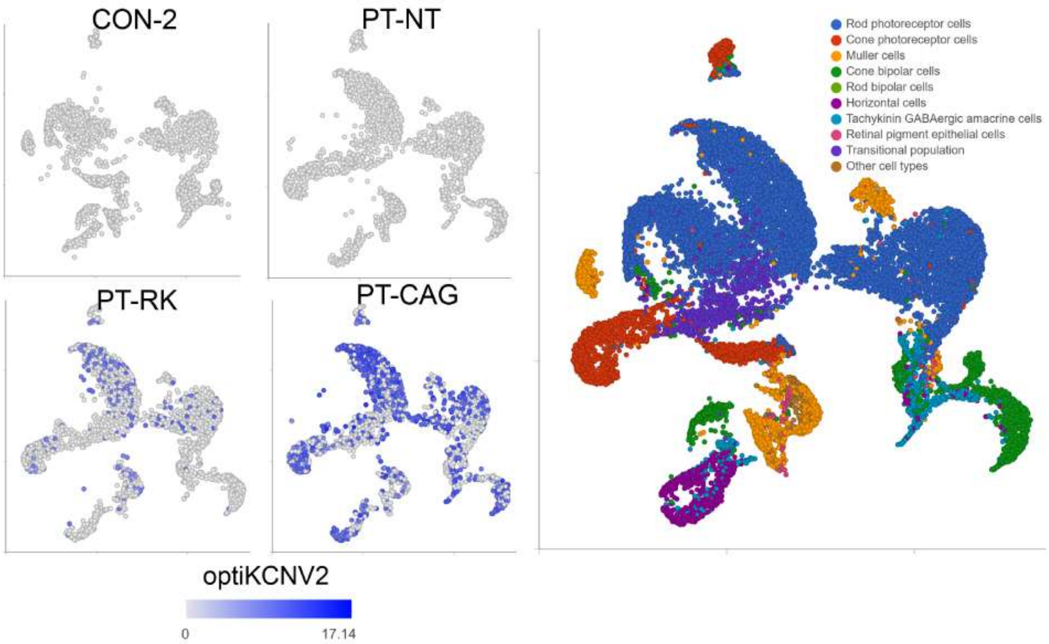

UMAP plots showing *optiKCNV2* expression in Control (CON-2) and *KCNV2* patient ROs transduced with RK-optiKCNV2 (PT-RK) or CAG-optiKCNV2 (PT-CAG) at 1.0E+11 VG/organoid, or non-transduced (PT-NT). The UMAP plot to the right is coloured by cell type cluster to aid the identification of each cell type.

Supplementary Table S1 – Disease Model DEGs

Differentially expressed genes between Control and KCNV2-deficient rods and cones.

DEGs in common between Control vs KCNV2 KO (CON-1 vs KO-NT) and Control vs KCNV2 patient (CON-2+3 vs PT-NT) comparisons. Differential expression analyses were performed for rods and cones separately. Note: Whilst all comparisons were performed on log_2(CPM+1) normalized counts, LSMean values are provided in counts per million (CPM) by the GSA algorithm in Partek Flow.

[Excel spreadsheet 'Supp Table S1 - Disease model DEGs']

Supplementary Table S2 – Enrichment results for disease model DEGs

Enrichment/over-representation analysis results for disease model DEGs. Results include Gene Ontology Biological Processes (GO_BP), GO Molecular Function (GO_MF), GO Cellular Component (GO_CC), and Reactome pathways. For rod photoreceptor Disease Model DEGs, all enrichment queries returned terms. For cone photoreceptor Disease Model DEGs, only GO_BP returned terms.

[Excel spreadsheet 'Supp Table S2 – Enrichment results for disease model DEGs']

Supplementary Table S3 – Restored genes

Genes restored by optiKCNV2 vectors.

A list of DEGs between Control and KCNV2-deficient (KO and patient) rods and cones that are restored by more than 20% upon transduction with opti*KCNV2* vectors. Note: Whilst all comparisons were performed on log_2(CPM+1) normalized counts, LSMean values are provided in counts per million (CPM) by the GSA algorithm in Partek Flow.

[Excel spreadsheet – 'Supp Table S3 - Restored genes']

Supplementary Table S4 – Enrichment results for restored genes.

Enrichment/over-representation analysis results for restored genes. The restored genes of rod photoreceptors returned terms for GO_BP only. No significantly enriched/over-represented results were returned for cone photoreceptor restored genes.

[Excel spreadsheet – 'Supp Table S4 – Enrichment results restored genes']

Supplementary Table S5. CRISPR/CAS9 gene editing guides and sequencing primers

Guide 1	GGAGACGGUCCUGGAGCUAC
Guide 2	GGGAGCCGGAACGGGCACCC
Guide 3	UACAUCGAGGAAGACGAAGA
Primer pair for mutational analysis	F: GCTGACTGCGTTCAGACCC<br>R: TGTCTGCTCCTCGTAGTCGT

Supplementary Table S6. QPCR primers and Taqman assays

Target (gene name)	Primer/probe details	Concentration
KCNV2 (wt*KCNV2*)	Hs00377936_m1	900nM (primer), 200nM (probe)
Opti*KCNV2*	FWD: ACGATGTGCCTAGCACCAACT<br>REV: CGGCCCACCACCAAGAG<br>Probe: CACCACCATTCCTC	900nM (primer), 200nM (probe)
GAPDH	Hs01060665_g1	900nM (primer), 200nM (probe)
ACTB (β-actin)	Hs02758991_g1	900nM (primer), 200nM (probe)

Supplementary Table S7. Antibodies

Target	Supplier	Cat.number	Species	Dilution IF/Wes
Kv8.2	Sigma	HPA031131	Rabbit	1:200/1:400(PLA)<br>1:40 WES
Kv2.1	Neuromab	Clone K89/34	Mouse	1:400/1:100 WES
Kv2.2	Neuromab	Clone N372B1	Mouse	1:400
PKCα	Invitrogen	MC5	Mouse	1:100
PDE6β	Invitrogen	PA1-722	Rabbit	1:300
Ribeye	BD Biosciences	612044	Mouse	1:400
RetGC	Protein tech	55127-1-AP	Rabbit	1:300
ABCA4	Abcam	3F4 ab77285	Mouse	1:100
Vglut	Synaptic systems	135 303	Rabbit	1:500
CRALBP/RLBP1	Sigma	Clone 1H7 WH0006017M1	Mouse	1:300
Rhodopsin (RHO)	Merck Millipore	MABN15	Mouse	1:500
Arrestin3 (ARR3)	Novusbio	NMP1-37003	Goat	1:300
L/M Opsin	Merck Millipore	AB5405	Rabbit	1:500
GAPDH	Synaptic systems	247 002	Rabbit	1:5000/ 1:10,000 (Wes)

Supplementary Table S8. Cell lines (Control, K (KCNV2 KO), Kp (KCNV2 patient) and independent differentiations (D) used in figures.

Figure	Experiment	Cell lines used
Figure 1D	Histology control vs. KCNV2 KO and patient IPSC day 150	K28 D2(KCNV2 KO)<br>K22p D2 (KCNV2 patient)<br>Control D136
Figure 1E	Wes analysis of Kv8.2 protein	Control<br>K28 (KCNV2 KO)<br>K22p (KCNV2 patient)
Figure 1F	QPCR analysis of KCNV2 mRNA	Control<br>K28, K12, K5 (KCNV2 KO)<br>K22p (KCNV2 patient; *n*= 3)
Figure 1 G	ONL thickness	Day 180 Control (D2,3,4,5)<br>Day 180 K28 (KCNV2 KO; D5 and 6)<br>Day 180 K12 (KCNV2 KO; D5 and 6)<br>Day 280 Control (D136)<br>Day 280 K28 (D3)
Figure 1H	Cone cell density analysis	Control (D4,5,2)<br>KCNV2 KO: K5 (D5, D1), K12 (D5,D6), K28 (D5,D6)
Figure 2B	IHC transduced organoids	Control<br>K28 (KCNV2 KO)
Figure 2C	IHC transduced organoids	Control<br>K28 (KCNV2 KO)
Figure 2D	IHC transduced organoids	Control (D2,3,4,5)<br>K28 (D5 and 6)<br>K12 (D5 and 6)
Figure 2E	PLA transduced organoids	Control<br>K12 (KCNV2 KO)
Figure 2F	Quantitative analysis of PLA in transduced organoids	K28 (D5 and D6)<br>K12 (D6)<br>Control (D7, D5,D2)
Figure 3 -5	scRNA-seq analysis of transduced organoids	Control<br>K28 (KCNV2 KO)<br>K22p (KCNV2 patient)
Supplementary 1A	Sequencing traces	Control<br>K22p (KCNV2 patient)<br>K28, K5, K12 (KCNV2 KO)

Supplementary 1E	IHC control and KCVN2 KO	Control<br>K28 (KCNV2 KO)
Supplementary 2A	IHC transduced organoids	Control<br>K28 (KCNV2 KOD5)
Supplementary 2C	IHC transduced organoids	Control<br>K22p (KCNV2 patient)
Supplementary 2D	QPCR transduced organoids	Control (D2,3,4,5)<br>KCNV2 KO: K12 (D5), K28 (D5), K28 (D6)
Supplementary 2E	QPCR transduced organoids	Control<br>K22p (KCNV2 patient)

Supplementary Table S9. Single cell RNA sequencing experimental information

**Project name**	**KCNV2 KO scRNA-seq**	**KCNV2 patient scRNA-seq**
**RO age at transduction**	244	176
**RO age at harvest (days)**	*KCNV2* KO (KO-NT, KO-RK, KO-CAG) = 279<br>Control (CON-1) = 283	*KCNV2* patient (PT-NT, PT-RK, PT-CAG) = 206<br>Control (CON-2, CON-3) = 201
**Treatment conditions**	Control: non-transduced<br>*KCNV2*-/- (KO or patient):<br>• Non-transduced<br>• AAV7m8-RK-optiKCNV2<br>• AAV7m8-CAG-optiKCNV2	
**AAV dose (VG per organoid)**	1.0E+11	
**Transduction duration**	5 weeks	
**10X Chromium kit chemistry**	3’ v3.1	5’ v2.0

Supplementary Table S10

SEQ ID NO	Name	Description	Sequence
1	AAVss-CAG-KCNV2-OPTI	AAV2 5’ ITR: 1-141 bp<br>CAG: 169-1901 bp<br>Kozak: 1926-1931 bp<br>KCNV2 (opti) 1932-3569 bp<br>WPREmut6: 3570-4180 bp<br>BGH pA: 4235-4442 bp<br>AAV2 3’ ITR: 4450-4590 bp	CCTGCAGGCAGCTGCGCGCTCGCTCGCTCACTGAGGC<br>CGCCCGGGCAAAGCCCGGGCGTCGGGCGACCTTTGGT<br>CGCCCGGCCTCAGTGAGCGAGCGAGCGCGCAGAGAG<br>GGAGTGGCCAACTCCATCACTAGGGGTTCCTTCTAGA<br>CAACTTTGTATAGAAAAGTTGCTCGACATTGATTATTG<br>ACTAGTTATTAATAGTAATCAATTACGGGGTCATTAG<br>TTCATAGCCCATATATGGAGTTCCGCGTTACATAACTT<br>ACGGTAAATGGCCCGCCTGGCTGACCGCCCAACGACC<br>CCCGCCCATTGACGTCAATAATGACGTATGTTCCCAT<br>AGTAACGCCAATAGGGACTTTCCATTGACGTCAATGG<br>GTGGAGTATTTACGGTAAACTGCCCACTTGGCAGTAC<br>ATCAAGTGTATCATATGCCAAGTACGCCCCCTATTGA<br>CGTCAATGACGGTAAATGGCCCGCCTGGCATTATGCC<br>CAGTACATGACCTTATGGGACTTTCCTACTTGGCAGTA<br>CATCTACGTATTAGTCATCGCTATTACCATGGTCGAGG<br>TGAGCCCCACGTTCTGCTTCACTCTCCCCATCTCCCCC<br>CCCTCCCCACCCCCAATTTTGTATTTATTTATTTTTTAA<br>TTATTTTGTGCAGCGATGGGGGCGGGGGGGGGGGGG<br>GGGCGCGCGCCAGGCGGGGCGGGGCGGGGCGAGGGG<br>CGGGGCGGGGCGAGGCGGAGAGGTGCGGCGGCAGCC<br>AATCAGAGCGGCGCGCTCCGAAAGTTTCCTTTTATGG<br>CGAGGCGGCGGCGGCGGCGGCCCTATAAAAAGCGAA<br>GCGCGCGGCGGGCGGGAGTCGCTGCGCGCTGCCTTCG<br>CCCCGTGCCCCGCTCCGCCGCCGCCTCGCGCCGCCCG<br>CCCCGGCTCTGACTGACCGCGTTACTCCCACAGGTGA<br>GCGGGCGGGACGGCCCTTCTCCTCCGGGCTGTAATTA<br>GCGCTTGGTTTAATGACGGCTTGTTTCTTTTCTGTGGC<br>TGCGTGAAAGCCTTGAGGGGCTCCGGGAGGGCCCTTT<br>GTGCGGGGGGAGCGGCTCGGGGGGTGCGTGCGTGTGT<br>GTGTGCGTGGGGAGCGCCGCGTGCGGCTCCGCGCTGC<br>CCGGCGGCTGTGAGCGCTGCGGGCGCGGCGCGGGGCT<br>TTGTGCGCTCCGCAGTGTGCGCGAGGGGAGCGCGGCC<br>GGGGGCGGTGCCCCGCGGTGCGGGGGGGGCTGCGAG<br>GGGAACAAAGGCTGCGTGCGGGGTGTGTGCGTGGGG<br>GGGTGAGCAGGGGGTGTGGGCGCGTCGGTCGGGCTG<br>CAACCCCCCCTGCACCCCCCTCCCCGAGTTGCTGAGC<br>ACGGCCCGGCTTCGGGTGCGGGGCTCCGTACGGGGCG<br>TGGCGCGGGGCTCGCCGTGCCGGGCGGGGGGTGGCG<br>GCAGGTGGGGGTGCCGGGCGGGGCGGGGCCGCCTCG<br>GGCCGGGGAGGGCTCGGGGGAGGGGCGCGGCGGCCC<br>CCGGAGCGCCGGCGGCTGTCGAGGCGCGGCGAGCCG<br>CAGCCATTGCCTTTTATGGTAATCGTGCGAGAGGGCG

			CAGGGACTTCCTTTGTCCCAAATCTGTGCGGAGCCGA<br>AATCTGGGAGGCGCCGCCGCACCCCCTCTAGCGGGCG<br>CGGGGCGAAGCGGTGCGGCGCCGGCAGGAAGGAAAT<br>GGGCGGGGAGGGCCTTCGTGCGTCGCCGCGCCGCCGT<br>CCCCTTCTCCCTCTCCAGCCTCGGGGCTGTCCGCGGGG<br>GGACGGCTGCCTTCGGGGGGGACGGGGCAGGGCGGG<br>GTTCGGCTTCTGGCGTGTGACCGGCGGCTCTAGAGCC<br>TCTGCTAACCATGTTCATGCCTTCTTCTTTTTCCTACAG<br>CTCCTGGGCAACGTGCTGGTTATTGTGCTGTCTCATCA<br>TTTTGGCAAAGAATTGCAAGTTTGTACAAAAAAGCAG<br>GCTGCCACCATGCTGAAGCAGAGCGAGAGAAGGCGG<br>AGCTGGTCCTACAGACCTTGGAACACCACAGAGAACG<br>AGGGCAGCCAGCACAGAAGATCCATCTGTTCTCTGGG<br>CGCCAGAAGCGGCTCTCAGGCCTCTATTCATGGCTGG<br>ACCGAGGGCAACTACAACTACTACATCGAAGAGGAC<br>GAGGACGGCGAGGAAGAGGACCAGTGGAAAGATGAC<br>CTGGCCGAGGAAGATCAGCAGGCCGGCGAAGTGACA<br>ACAGCCAAGCCTGAAGGACCTAGCGATCCTCCTGCTC<br>TGCTGAGCACCCTGAATGTGAATGTCGGCGGCCACAG<br>CTACCAGCTGGATTACTGTGAACTGGCCGGCTTTCCC<br>AAGACCAGACTGGGCAGACTGGCCACCAGCACAAGC<br>AGATCTAGACAGCTGAGCCTGTGCGACGACTACGAGG<br>AACAGACCGACGAGTACTTCTTCGACAGAGATCCCGC<br>CGTGTTTCAGCTGGTGTACAACTTCTACCTGAGCGGC<br>GTGCTGCTGGTGCTGGATGGACTGTGCCCTCGGAGAT<br>TTCTGGAAGAACTCGGCTACTGGGGCGTCAGACTGAA<br>GTACACCCCTCGGTGCTGCCGGATCTGCTTCGAGGAA<br>AGAAGGGACGAGCTGAGCGAGCGGCTGAAGATCCAG<br>CATGAACTGAGAGCCCAGGCTCAGGTGGAAGAGGCC<br>GAAGAACTGTTCCGGGACATGAGATTCTACGGCCCTC<br>AACGGCGGAGACTGTGGAACCTGATGGAAAAGCCTTT<br>TAGCAGCGTGGCCGCCAAGGCCATTGGAGTGGCCTCT<br>TCTACATTCGTGCTGGTGTCTGTGGTGGCCCTGGCTCT<br>GAATACCGTGGAAGAGATGCAGCAGCACTCTGGCCA<br>AGGCGAAGGCGGACCTGATCTGAGGCCTATCCTGGAA<br>CACGTGGAAATGCTGTGCATGGGCTTTTTCACCCTGG<br>AATACCTGCTGCGGCTGGCCTCTACACCCGACCTGAG<br>AAGATTTGCCAGATCTGCCCTGAACCTGGTGGATCTG<br>GTGGCTATCCTGCCTCTGTATCTGCAGCTGCTGCTGGA<br>ATGTTTTACCGGCGAGGGACATCAGAGGGGCCAGACA<br>GTGGGATCTGTGGGCAAAGTTGGACAGGTGCTGAGAG<br>TGATGCGGCTGATGAGAATCTTCCGGATCCTGAAGCT<br>GGCCAGACACAGCACCGGACTGAGAGCTTTCGGCTTC<br>ACCCTGAGACAGTGCTACCAGCAAGTGGGCTGCCTGC<br>TGCTGTTTATCGCCATGGGCATCTTCACCTTCTCTGCC<br>GCCGTGTACAGCGTGGAACACGATGTGCCTAGCACCA<br>ACTTCACCACCATTCCTCACTCTTGGTGGTGGGCCGCT

			GTGTCTATCTCTACAGTCGGCTACGGCGACATGTACC<br>CAGAGACACACCTGGGCAGATTCTTCGCCTTCCTGTG<br>TATCGCCTTCGGCATCATCCTGAACGGCATGCCCATC<br>AGCATCCTGTACAACAAGTTCAGCGACTACTACAGCA<br>AGCTCAAGGCCTACGAGTACACCACAATTCGGAGAGA<br>GCGGGGCGAAGTCAACTTCATGCAGCGGGCCAGAAA<br>GAAAATCGCCGAGTGCCTGCTGGGCAGCAATCCTCAG<br>CTGACCCCTCGGCAAGAGAACTGACGATTTCTGGATC<br>CACGCTAGCAATCAACCTCTGGATTACAAAATTTGTG<br>AAAGATTGACTGGTATTCTTAACTATGTTGCTCCTTTT<br>ACGCTATGTGGATACGCTGCTTTAATGCCTTTGTATCA<br>TGCTATTGCTTCCCGTATGGCTTTCATTTTCTCCTCCTT<br>GTATAAATCCTGGTTGCTGTCTCTTTATGAGGAGTTGT<br>GGCCCGTTGTCAGGCAACGTGGCGTGGTGTGCACTGT<br>GTTTGCTGACGCAACCCCCACTGGTTGGGGCATTGCC<br>ACCACCTGTCAGCTCCTTTCCGGGACTTTCGCTTTCCC<br>CCTCCCTATTGCCACGGCGGAACTCATCGCCGCCTGC<br>CTTGCCCGCTGCTGGACAGGGGCTCGGCTGTTGGGCA<br>CTGACAATTCCGTGGTGTTGTCGGGGAAATCATCGTC<br>CTTTCCTTGGCTGCTCGCCTGTGTTGCCACCTGGATTC<br>TGCGCGGGACGTCCTTCTGCTACGTCCCTTCGGCCCTC<br>AATCCAGCGGACCTTCCTTCCCGCGGCCTGCTGCCGG<br>CTCTGCGGCCTCTTCCGCGTCTTCGCCTTCGCCCTCAG<br>ACGAGTCGGATCTCCCTTTGGGCCGCCTCCCCGCACC<br>CAGCTTTCTTGTACAAAGTGGGAATTCCTAGAGCTCG<br>CTGATCAGCCTCGACTGTGCCTTCTAGTTGCCAGCCAT<br>CTGTTGTTTGCCCCTCCCCCGTGCCTTCCTTGACCCTG<br>GAAGGTGCCACTCCCACTGTCCTTTCCTAATAAAATG<br>AGGAAATTGCATCGCATTGTCTGAGTAGGTGTCATTC<br>TATTCTGGGGGGTGGGGTGGGGCAGGACAGCAAGGG<br>GGAGGATTGGGAAGAGAATAGCAGGCATGCTGGGGA<br>GGGCCGCAGGAACCCCTAGTGATGGAGTTGGCCACTC<br>CCTCTCTGCGCGCTCGCTCGCTCACTGAGGCCGGGCG<br>ACCAAAGGTCGCCCGACGCCCGGGCTTTGCCCGGGCG<br>GCCTCAGTGAGCGAGCGAGCGCGCAGCTGCCTGCAGG<br>GGCGCCTGATGCGGTATTTTCTCCTTACGCATCTGTGC<br>GGTATTTCACACCGCATACGTCAAAGCAACCATAGTA<br>CGCGCCCTGTAGCGGCGCATTAAGCGCGGCGGGTGTG<br>GTGGTTACGCGCAGCGTGACCGCTACACTTGCCAGCG<br>CCTTAGCGCCCGCTCCTTTCGCTTTCTTCCCTTCCTTTC<br>TCGCCACGTTCGCCGGCTTTCCCCGTCAAGCTCTAAAT<br>CGGGGGCTCCCTTTAGGGTTCCGATTTAGTGCTTTACG<br>GCACCTCGACCCCAAAAAACTTGATTTGGGTGATGGT<br>TCACGTAGTGGGCCATCGCCCTGATAGACGGTTTTTC<br>GCCCTTTGACGTTGGAGTCCACGTTCTTTAATAGTGGA<br>CTCTTGTTCCAAACTGGAACAACACTCAACTCTATCTC<br>GGGCTATTCTTTTGATTTATAAGGGATTTTGCCGATTT

			CGGTCTATTGGTTAAAAAATGAGCTGATTTAACAAAA<br>ATTTAACGCGAATTTTAACAAAATATTAACGTTTACA<br>ATTTTATGGTGCACTCTCAGTACAATCTGCTCTGATGC<br>CGCATAGTTAAGCCAGCCCCGACACCCGCCAACACCC<br>GCTGACGCGCCCTGACGGGCTTGTCTGCTCCCGGCAT<br>CCGCTTACAGACAAGCTGTGACCGTCTCCGGGAGCTG<br>CATGTGTCAGAGGTTTTCACCGTCATCACCGAAACGC<br>GCGAGACGAAAGGGCCTCGTGATACGCCTATTTTTAT<br>AGGTTAATGTCATGATAATAATGGTTTCTTAGACGTCC<br>TGGCCCGTGTCTCAAAATCTCTGATGTTACATTGCACA<br>AGATAAAAATATATCATCATGAACAATAAAACTGTCT<br>GCTTACATAAACAGTAATACAAGGGGTGTTATGAGCC<br>ATATTCAACGGGAAACGTCGAGGCCGCGATTAAATTC<br>CAACATGGATGCTGATTTATATGGGTATAAATGGGCT<br>CGCGATAATGTCGGGCAATCAGGTGCGACAATCTATC<br>GCTTGTATGGGAAGCCCGATGCGCCAGAGTTGTTTCT<br>GAAACATGGCAAAGGTAGCGTTGCCAATGATGTTACA<br>GATGAGATGGTCAGACTAAACTGGCTGACGGAATTTA<br>TGCCTCTTCCGACCATCAAGCATTTTATCCGTACTCCT<br>GATGATGCATGGTTACTCACCACTGCGATCCCCGGAA<br>AAACAGCATTCCAGGTATTAGAAGAATATCCTGATTC<br>AGGTGAAAATATTGTTGATGCGCTGGCAGTGTTCCTG<br>CGCCGGTTGCATTCGATTCCTGTTTGTAATTGTCCTTT<br>TAACAGCGATCGCGTATTTCGTCTCGCTCAGGCGCAA<br>TCACGAATGAATAACGGTTTGGTTGATGCGAGTGATT<br>TTGATGACGAGCGTAATGGCTGGCCTGTTGAACAAGT<br>CTGGAAAGAAATGCATAAACTTTTGCCATTCTCACCG<br>GATTCAGTCGTCACTCATGGTGATTTCTCACTTGATAA<br>CCTTATTTTTGACGAGGGGAAATTAATAGGTTGTATTG<br>ATGTTGGACGAGTCGGAATCGCAGACCGATACCAGGA<br>TCTTGCCATCCTATGGAACTGCCTCGGTGAGTTTTCTC<br>CTTCATTACAGAAACGGCTTTTTCAAAAATATGGTATT<br>GATAATCCTGATATGAATAAATTGCAGTTTCATTTGAT<br>GCTCGATGAGTTTTTCTAATCAGAATTGGTTAATTGGT<br>TGTAACACTGGCAGAGCATTACGCTGACTTGACGGGA<br>CGGCGCAAGCTCATGACCAAAATCCCTTAACGTGAGT<br>TACGCGTGAAGATCCTTTTTGATAATCTCATGACCAA<br>AATCCCTTAACGTGAGTTTTCGTTCCACTGAGCGTCAG<br>ACCCCGTAGAAAAGATCAAAGGATCTTCTTGAGATCC<br>TTTTTTTCTGCGCGTAATCTGCTGCTTGCAAACAAAAA<br>AACCACCGCTACCAGCGGTGGTTTGTTTGCCGGATCA<br>AGAGCTACCAACTCTTTTTCCGAAGGTAACTGGCTTC<br>AGCAGAGCGCAGATACCAAATACTGTTCTTCTAGTGT<br>AGCCGTAGTTAGGCCACCACTTCAAGAACTCTGTAGC<br>ACCGCCTACATACCTCGCTCTGCTAATCCTGTTACCAG<br>TGGCTGCTGCCAGTGGCGATAAGTCGTGTCTTACCGG<br>GTTGGACTCAAGACGATAGTTACCGGATAAGGCGCAG

			CGGTCGGGCTGAACGGGGGGTTCGTGCACACAGCCCA<br>GCTTGGAGCGAACGACCTACACCGAACTGAGATACCT<br>ACAGCGTGAGCTATGAGAAAGCGCCACGCTTCCCGAA<br>GGGAGAAAGGCGGACAGGTATCCGGTAAGCGGCAGG<br>GTCGGAACAGGAGAGCGCACGAGGGAGCTTCCAGGG<br>GGAAACGCCTGGTATCTTTATAGTCCTGTCGGGTTTCG<br>CCACCTCTGACTTGAGCGTCGATTTTTGTGATGCTCGT<br>CAGGGGGGCGGAGCCTATGGAAAAACGCCAGCAACG<br>CGGCCTTTTTACGGTTCCTGGCCTTTTGCTGGCCTTTT<br>GCTCACATGT
2	AAVss-<br>CAG-<br>KCNV2<br>-WT	AAV2 5' ITR: 1-141 bp<br>CAG: 169-1901 bp<br>Kozak: 1926-1931 bp<br>KCNV2 (WT) 1932-3569 bp<br>WPREmut6 : 3570-4180 bp<br>BGH pA: 4235-4442 bp<br>AAV2 3' ITR: 4450-4590 bp	CCTGCAGGCAGCTGCGCGCTCGCTCGCTCACTGAGGC<br>CGCCCGGGCAAAGCCCGGGCGTCGGGCGACCTTTGGT<br>CGCCCGGCCTCAGTGAGCGAGCGAGCGCGCAGAGAG<br>GGAGTGGCCAACTCCATCACTAGGGGTTCCTTCTAGA<br>CAACTTTGTATAGAAAAGTTGCTCGACATTGATTATTG<br>ACTAGTTATTAATAGTAATCAATTACGGGGTCATTAG<br>TTCATAGCCCATATATGGAGTTCCGCGTTACATAACTT<br>ACGGTAAATGGCCCGCCTGGCTGACCGCCCAACGACC<br>CCCGCCCATTGACGTCAATAATGACGTATGTTCCCAT<br>AGTAACGCCAATAGGGACTTTCCATTGACGTCAATGG<br>GTGGAGTATTTACGGTAAACTGCCCACTTGGCAGTAC<br>ATCAAGTGTATCATATGCCAAGTACGCCCCCTATTGA<br>CGTCAATGACGGTAAATGGCCCGCCTGGCATTATGCC<br>CAGTACATGACCTTATGGGACTTTCCTACTTGGCAGTA<br>CATCTACGTATTAGTCATCGCTATTACCATGGTCGAGG<br>TGAGCCCCACGTTCTGCTTCACTCTCCCCATCTCCCCC<br>CCCTCCCCACCCCCAATTTTGTATTTATTTATTTTTTAA<br>TTATTTTGTGCAGCGATGGGGGCGGGGGGGGGGGGG<br>GGGCGCGCGCCAGGCGGGGCGGGGCGGGGCGAGGGG<br>CGGGGCGGGGCGAGGCGGAGAGGTGCGGCGGCAGCC<br>AATCAGAGCGGCGCGCTCCGAAAGTTTCCTTTTATGG<br>CGAGGCGGCGGCGGCGGCGGCCCTATAAAAAGCGAA<br>GCGCGCGGCGGGCGGGAGTCGCTGCGCGCTGCCTTCG<br>CCCCGTGCCCCGCTCCGCCGCCGCCTCGCGCCGCCCG<br>CCCCGGCTCTGACTGACCGCGTTACTCCCACAGGTGA<br>GCGGGCGGGACGGCCCTTCTCCTCCGGGCTGTAATTA<br>GCGCTTGGTTTAATGACGGCTTGTTTCTTTTCTGTGGC<br>TGCGTGAAAGCCTTGAGGGGCTCCGGGAGGGCCCTTT<br>GTGCGGGGGGAGCGGCTCGGGGGGTGCGTGCGTGTGT<br>GTGTGCGTGGGGAGCGCCGCGTGCGGCTCCGCGCTGC<br>CCGGCGGCTGTGAGCGCTGCGGGCGCGGCGCGGGGCT<br>TTGTGCGCTCCGCAGTGTGCGCGAGGGGAGCGCGGCC<br>GGGGGCGGTGCCCCGCGGTGCGGGGGGGGCTGCGAG<br>GGGAACAAAGGCTGCGTGCGGGGTGTGTGCGTGGGG<br>GGGTGAGCAGGGGGTGTGGGCGCGTCGGTCGGGCTG<br>CAACCCCCCCTGCACCCCCCTCCCCGAGTTGCTGAGC<br>ACGGCCCGGCTTCGGGTGCGGGGCTCCGTACGGGGCG<br>TGGCGCGGGGCTCGCCGTGCCGGGCGGGGGGTGGCG<br>GCAGGTGGGGGTGCCGGGCGGGGCGGGGCCGCCTCG<br>GGCCGGGGAGGGCTCGGGGGAGGGGCGCGGCGGCCC

			CCGGAGCGCCGGCGGCTGTCGAGGCGCGGCGAGCCG<br>CAGCCATTGCCTTTTATGGTAATCGTGCGAGAGGGCG<br>CAGGGACTTCCTTTGTCCCAAATCTGTGCGGAGCCGA<br>AATCTGGGAGGCGCCGCCGCACCCCCTCTAGCGGGCG<br>CGGGGCGAAGCGGTGCGGCGCCGGCAGGAAGGAAAT<br>GGGCGGGGAGGGCCTTCGTGCGTCGCCGCGCCGCCGT<br>CCCCTTCTCCCTCTCCAGCCTCGGGGCTGTCCGCGGGG<br>GGACGGCTGCCTTCGGGGGGGACGGGGCAGGGCGGG<br>GTTCGGCTTCTGGCGTGTGACCGGCGGCTCTAGAGCC<br>TCTGCTAACCATGTTCATGCCTTCTTCTTTTTCCTACAG<br>CTCCTGGGCAACGTGCTGGTTATTGTGCTGTCTCATCA<br>TTTTGGCAAAGAATTGCAAGTTTGTACAAAAAAGCAG<br>GCTGCCACCATGCTCAAACAGAGTGAGAGGAGACGG<br>TCCTGGAGCTACAGGCCCTGGAACACGACGGAGAATG<br>AGGGCAGCCAACACCGCAGGAGCATTTGCTCCCTGGG<br>TGCCCGTTCCGGCTCCCAGGCCAGCATCCACGGCTGG<br>ACAGAGGGCAACTATAACTACTACATCGAGGAAGAC<br>GAAGACGGCGAGGAGGAGGACCAGTGGAAGGACGAC<br>CTGGCAGAAGAGGACCAGCAGGCAGGGGAGGTCACC<br>ACCGCCAAGCCCGAGGGCCCCAGCGACCCTCCGGCCC<br>TGCTGTCCACGCTGAATGTGAACGTGGGTGGCCACAG<br>CTACCAGCTGGACTACTGCGAGCTGGCCGGCTTCCCC<br>AAGACGCGCCTAGGTCGCCTGGCCACCTCCACCAGCC<br>GCAGCCGCCAGCTAAGCCTGTGCGACGACTACGAGGA<br>GCAGACAGACGAATACTTCTTCGACCGCGACCCGGCC<br>GTCTTCCAGCTGGTCTACAATTTCTACCTGTCCGGGGT<br>GCTGCTGGTGCTCGACGGGCTGTGTCCGCGCCGCTTC<br>CTGGAGGAGCTGGGCTACTGGGGCGTGCGGCTCAAGT<br>ACACGCCACGCTGCTGCCGCATCTGCTTCGAGGAGCG<br>GCGCGACGAGCTGAGCGAACGGCTCAAGATCCAGCA<br>CGAGCTGCGCGCGCAGGCGCAGGTCGAGGAGGCGGA<br>GGAACTCTTCCGCGACATGCGCTTCTACGGCCCGCAG<br>CGGCGCCGCCTCTGGAACCTCATGGAGAAGCCATTCT<br>CCTCGGTGGCCGCCAAGGCCATCGGGGTGGCCTCCAG<br>CACCTTCGTGCTCGTCTCCGTGGTGGCGCTGGCGCTCA<br>ACACCGTGGAGGAGATGCAGCAGCACTCGGGGCAGG<br>GCGAGGGCGGCCCAGACCTGCGGCCCATCCTGGAGCA<br>CGTGGAGATGCTGTGCATGGGCTTCTTCACGCTCGAG<br>TACCTGCTGCGCCTAGCCTCCACGCCCGACCTGAGGC<br>GCTTCGCGCGCAGCGCCCTCAACCTGGTGGACCTGGT<br>GGCCATCCTGCCGCTCTACCTTCAGCTGCTGCTCGAGT<br>GCTTCACGGGCGAGGGCCACCAACGCGGCCAGACGG<br>TGGGCAGCGTGGGTAAGGTGGGTCAGGTGTTGCGCGT<br>CATGCGCCTCATGCGCATCTTCCGCATCCTCAAGCTGG<br>CGCGCCACTCCACCGGACTGCGTGCCTTCGGCTTCAC<br>GCTGCGCCAGTGCTACCAGCAGGTGGGCTGCCTGCTG<br>CTCTTCATCGCCATGGGCATCTTCACTTTCTCTGCGGC<br>TGTCTACTCTGTGGAGCACGATGTGCCCAGCACCAAC<br>TTCACTACCATCCCCCACTCCTGGTGGTGGGCCGCGGT<br>GAGCATCTCCACCGTGGGCTACGGAGACATGTACCCA<br>GAGACCCACCTGGGCAGGTTTTTTGCCTTCCTCTGCAT

			TGCTTTTGGGATCATTCTCAACGGGATGCCCATTTCCA TCCTCTACAACAAGTTTTCTGATTACTACAGCAAGCTG AAGGCTTATGAGTATACCACCATACGCAGGGAGAGG GGAGAGGTGAACTTCATGCAGAGAGCCAGAAAGAAG ATAGCTGAGTGTTTGCTTGGAAGCAACCCACAGCTCA CCCCAAGACAAGAGAATTAGCGATTTCTGGATCCACG CTAGCAATCAACCTCTGGATTACAAAATTTGTGAAAG ATTGACTGGTATTCTTAACTATGTTGCTCCTTTTACGC TATGTGGATACGCTGCTTTAATGCCTTTGTATCATGCT ATTGCTTCCCGTATGGCTTTCATTTTCTCCTCCTTGTAT AAATCCTGGTTGCTGTCTCTTTATGAGGAGTTGTGGCC CGTTGTCAGGCAACGTGGCGTGGTGTGCACTGTGTTT GCTGACGCAACCCCCACTGGTTGGGGCATTGCCACCA CCTGTCAGCTCCTTTCCGGGACTTTCGCTTTCCCCCTC CCTATTGCCACGGCGGAACTCATCGCCGCCTGCCTTG CCCGCTGCTGGACAGGGGCTCGGCTGTTGGGCACTGA CAATTCCGTGGTGTTGTCGGGGAAATCATCGTCCTTTC CTTGGCTGCTCGCCTGTGTTGCCACCTGGATTCTGCGC GGGACGTCCTTCTGCTACGTCCCTTCGGCCCTCAATCC AGCGGACCTTCCTTCCCGCGGCCTGCTGCCGGCTCTGC GGCCTCTTCCGCGTCTTCGCCTTCGCCCTCAGACGAGT CGGATCTCCCTTTGGGCCGCCTCCCCGCACCCAGCTTT CTTGTACAAAGTGGGAATTCCTAGAGCTCGCTGATCA GCCTCGACTGTGCCTTCTAGTTGCCAGCCATCTGTTGT TTGCCCCTCCCCCGTGCCTTCCTTGACCCTGGAAGGTG CCACTCCCACTGTCCTTTCCTAATAAAATGAGGAAATT GCATCGCATTGTCTGAGTAGGTGTCATTCTATTCTGGG GGGTGGGGTGGGGCAGGACAGCAAGGGGGAGGATTG GGAAGAGAATAGCAGGCATGCTGGGGAGGGCCGCAG GAACCCCTAGTGATGGAGTTGGCCACTCCCTCTCTGC GCGCTCGCTCGCTCACTGAGGCCGGGCGACCAAAGGT CGCCCGACGCCCGGGCTTTGCCCGGGCGGCCTCAGTG AGCGAGCGAGCGCGCAGCTGCCTGCAGGGGCGCCTG ATGCGGTATTTTCTCCTTACGCATCTGTGCGGTATTTC ACACCGCATACGTCAAAGCAACCATAGTACGCGCCCT GTAGCGGCGCATTAAGCGCGGCGGGTGTGGTGGTTAC GCGCAGCGTGACCGCTACACTTGCCAGCGCCTTAGCG CCCGCTCCTTTCGCTTTCTTCCCTTCCTTTCTCGCCACG TTCGCCGGCTTTCCCCGTCAAGCTCTAAATCGGGGGCT CCCTTTAGGGTTCCGATTTAGTGCTTTACGGCACCTCG ACCCCAAAAAACTTGATTTGGGTGATGGTTCACGTAG TGGGCCATCGCCCTGATAGACGGTTTTTCGCCCTTTGA CGTTGGAGTCCACGTTCTTTAATAGTGGACTCTTGTTC CAAACTGGAACAACACTCAACTCTATCTCGGGCTATT CTTTTGATTTATAAGGGATTTTGCCGATTTCGGTCTAT TGGTTAAAAAATGAGCTGATTTAACAAAAATTTAACG CGAATTTTAACAAAATATTAACGTTTACAATTTTATGG TGCACTCTCAGTACAATCTGCTCTGATGCCGCATAGTT AAGCCAGCCCCGACACCCGCCAACACCCGCTGACGCG CCCTGACGGGCTTGTCTGCTCCCGGCATCCGCTTACAG ACAAGCTGTGACCGTCTCCGGGAGCTGCATGTGTCAG

			AGGTTTTCACCGTCATCACCGAAACGCGCGAGACGAA<br>AGGGCCTCGTGATACGCCTATTTTTATAGGTTAATGTC<br>ATGATAATAATGGTTTCTTAGACGTCCTGGCCCGTGTC<br>TCAAAATCTCTGATGTTACATTGCACAAGATAAAAAT<br>ATATCATCATGAACAATAAAACTGTCTGCTTACATAA<br>ACAGTAATACAAGGGGTGTTATGAGCCATATTCAACG<br>GGAAACGTCGAGGCCGCGATTAAATTCCAACATGGAT<br>GCTGATTTATATGGGTATAAATGGGCTCGCGATAATG<br>TCGGGCAATCAGGTGCGACAATCTATCGCTTGTATGG<br>GAAGCCCGATGCGCCAGAGTTGTTTCTGAAACATGGC<br>AAAGGTAGCGTTGCCAATGATGTTACAGATGAGATGG<br>TCAGACTAAACTGGCTGACGGAATTTATGCCTCTTCC<br>GACCATCAAGCATTTTATCCGTACTCCTGATGATGCAT<br>GGTTACTCACCACTGCGATCCCCGGAAAAACAGCATT<br>CCAGGTATTAGAAGAATATCCTGATTCAGGTGAAAAT<br>ATTGTTGATGCGCTGGCAGTGTTCCTGCGCCGGTTGCA<br>TTCGATTCCTGTTTGTAATTGTCCTTTTAACAGCGATC<br>GCGTATTTCGTCTCGCTCAGGCGCAATCACGAATGAA<br>TAACGGTTTGGTTGATGCGAGTGATTTTGATGACGAG<br>CGTAATGGCTGGCCTGTTGAACAAGTCTGGAAAGAAA<br>TGCATAAACTTTTGCCATTCTCACCGGATTCAGTCGTC<br>ACTCATGGTGATTTCTCACTTGATAACCTTATTTTTGA<br>CGAGGGGAAATTAATAGGTTGTATTGATGTTGGACGA<br>GTCGGAATCGCAGACCGATACCAGGATCTTGCCATCC<br>TATGGAACTGCCTCGGTGAGTTTTCTCCTTCATTACAG<br>AAACGGCTTTTTCAAAAATATGGTATTGATAATCCTG<br>ATATGAATAAATTGCAGTTTCATTTGATGCTCGATGA<br>GTTTTTCTAATCAGAATTGGTTAATTGGTTGTAACACT<br>GGCAGAGCATTACGCTGACTTGACGGGACGGCGCAA<br>GCTCATGACCAAAATCCCTTAACGTGAGTTACGCGTG<br>AAGATCCTTTTTGATAATCTCATGACCAAAATCCCTTA<br>ACGTGAGTTTTCGTTCCACTGAGCGTCAGACCCCGTA<br>GAAAAGATCAAAGGATCTTCTTGAGATCCTTTTTTTCT<br>GCGCGTAATCTGCTGCTTGCAAACAAAAAAACCACCG<br>CTACCAGCGGTGGTTTGTTTGCCGGATCAAGAGCTAC<br>CAACTCTTTTTCCGAAGGTAACTGGCTTCAGCAGAGC<br>GCAGATACCAAATACTGTTCTTCTAGTGTAGCCGTAG<br>TTAGGCCACCACTTCAAGAACTCTGTAGCACCGCCTA<br>CATACCTCGCTCTGCTAATCCTGTTACCAGTGGCTGCT<br>GCCAGTGGCGATAAGTCGTGTCTTACCGGGTTGGACT<br>CAAGACGATAGTTACCGGATAAGGCGCAGCGGTCGG<br>GCTGAACGGGGGGTTCGTGCACACAGCCCAGCTTGGA<br>GCGAACGACCTACACCGAACTGAGATACCTACAGCGT<br>GAGCTATGAGAAAGCGCCACGCTTCCCGAAGGGAGA<br>AAGGCGGACAGGTATCCGGTAAGCGGCAGGGTCGGA<br>ACAGGAGAGCGCACGAGGGAGCTTCCAGGGGGAAAC<br>GCCTGGTATCTTTATAGTCCTGTCGGGTTTCGCCACCT<br>CTGACTTGAGCGTCGATTTTTGTGATGCTCGTCAGGGG<br>GGCGGAGCCTATGGAAAAACGCCAGCAACGCGGCCT<br>TTTTACGGTTCCTGGCCTTTTGCTGGCCTTTTGCTCAC<br>ATGT

3	AAVss-RK-KCNV2-OPTI	AAV2 5' ITR: 1-141 bp<br>RK: 169-788 bp<br>Kozak: 813-818 bp<br>KCNV2(Opti): 819-2456 bp<br>WPRE(mut 6): 2457-3067 bp<br>BGH pA: 3122-3329 bp<br>AAV2 3' ITR: 3337-3477 bp	CCTGCAGGCAGCTGCGCGCTCGCTCGCTCACTGAGGC<br>CGCCCGGGCAAAGCCCGGGCGTCGGGCGACCTTTGGT<br>CGCCCGGCCTCAGTGAGCGAGCGAGCGCGCAGAGAG<br>GGAGTGGCCAACTCCATCACTAGGGGTTCCTTCTAGA<br>CAACTTTGTATAGAAAAGTTGTGTAGTTAATGATTAA<br>CCCGCCATGCTACTTATCTACGTACATTTATATTGGCT<br>CATGTCCAACATTACCGCCATGTTGACATTGATTATTG<br>ACTAGAATTCGCTAGCAAGATCCAAGCTCAGATCTCG<br>ATCGAGTTGGGCCCCAGAAGCCTGGTGGTTGTTTGTC<br>CTTCTCAGGGGAAAAGTGAGGCGGCCCCTTGGAGGAA<br>GGGGCCGGGCAGAATGATCTAATCGGATTCCAAGCAG<br>CTCAGGGGATTGTCTTTTTCTAGCACCTTCTTGCCACT<br>CCTAAGCGTCCTCCGTGACCCCGGCTGGGATTTAGCC<br>TGGTGCTGTGTCAGCCCCGGTCTCCCAGGGGCTTCCC<br>AGTGGTCCCCAGGAACCCTCGACAGGGCCCGGTCTCT<br>CTCGTCCAGCAAGGGCAGGGACGGGCCACAGGCCAA<br>GGGCCCTCGATCGAGGAACTGAAAAACCAGAAAGTT<br>AACTGGTAAGTTTAGTCTTTTTGTCTTTTATTTCAGGT<br>CCCGGATCCGGTGGTGGTGCAAATCAAAGAACTGCTC<br>CTCAGTGGATGTTGCCTTTACTTCTAGGCCTGTACGGA<br>AGTGTTACTTCTGCTCTAAAAGCTGCGGAATTGTACCC<br>GCGGCCGCCAAGTTTGTACAAAAAAGCAGGCTGCCAC<br>CATGCTGAAGCAGAGCGAGAGAAGGCGGAGCTGGTC<br>CTACAGACCTTGGAACACCACAGAGAACGAGGGCAG<br>CCAGCACAGAAGATCCATCTGTTCTCTGGGCGCCAGA<br>AGCGGCTCTCAGGCCTCTATTCATGGCTGGACCGAGG<br>GCAACTACAACTACTACATCGAAGAGGACGAGGACG<br>GCGAGGAAGAGGACCAGTGGAAAGATGACCTGGCCG<br>AGGAAGATCAGCAGGCCGGCGAAGTGACAACAGCCA<br>AGCCTGAAGGACCTAGCGATCCTCCTGCTCTGCTGAG<br>CACCCTGAATGTGAATGTCGGCGGCCACAGCTACCAG<br>CTGGATTACTGTGAACTGGCCGGCTTTCCCAAGACCA<br>GACTGGGCAGACTGGCCACCAGCACAAGCAGATCTA<br>GACAGCTGAGCCTGTGCGACGACTACGAGGAACAGA<br>CCGACGAGTACTTCTTCGACAGAGATCCCGCCGTGTT<br>TCAGCTGGTGTACAACTTCTACCTGAGCGGCGTGCTG<br>CTGGTGCTGGATGGACTGTGCCCTCGGAGATTTCTGG<br>AAGAACTCGGCTACTGGGGCGTCAGACTGAAGTACAC<br>CCCTCGGTGCTGCCGGATCTGCTTCGAGGAAAGAAGG<br>GACGAGCTGAGCGAGCGGCTGAAGATCCAGCATGAA<br>CTGAGAGCCCAGGCTCAGGTGGAAGAGGCCGAAGAA<br>CTGTTCCGGGACATGAGATTCTACGGCCCTCAACGGC<br>GGAGACTGTGGAACCTGATGGAAAAGCCTTTTAGCAG<br>CGTGGCCGCCAAGGCCATTGGAGTGGCCTCTTCTACA<br>TTCGTGCTGGTGTCTGTGGTGGCCCTGGCTCTGAATAC<br>CGTGGAAGAGATGCAGCAGCACTCTGGCCAAGGCGA<br>AGGCGGACCTGATCTGAGGCCTATCCTGGAACACGTG<br>GAAATGCTGTGCATGGGCTTTTTCACCCTGGAATACCT<br>GCTGCGGCTGGCCTCTACACCCGACCTGAGAAGATTT<br>GCCAGATCTGCCCTGAACCTGGTGGATCTGGTGGCTA<br>TCCTGCCTCTGTATCTGCAGCTGCTGCTGGAATGTTTT

			ACCGGCGAGGGACATCAGAGGGGCCAGACAGTGGGA<br>TCTGTGGGCAAAGTTGGACAGGTGCTGAGAGTGATGC<br>GGCTGATGAGAATCTTCCGGATCCTGAAGCTGGCCAG<br>ACACAGCACCGGACTGAGAGCTTTCGGCTTCACCCTG<br>AGACAGTGCTACCAGCAAGTGGGCTGCCTGCTGCTGT<br>TTATCGCCATGGGCATCTTCACCTTCTCTGCCGCCGTG<br>TACAGCGTGGAACACGATGTGCCTAGCACCAACTTCA<br>CCACCATTCCTCACTCTTGGTGGTGGGCCGCTGTGTCT<br>ATCTCTACAGTCGGCTACGGCGACATGTACCCAGAGA<br>CACACCTGGGCAGATTCTTCGCCTTCCTGTGTATCGCC<br>TTCGGCATCATCCTGAACGGCATGCCCATCAGCATCC<br>TGTACAACAAGTTCAGCGACTACTACAGCAAGCTCAA<br>GGCCTACGAGTACACCACAATTCGGAGAGAGCGGGG<br>CGAAGTCAACTTCATGCAGCGGGCCAGAAAGAAAAT<br>CGCCGAGTGCCTGCTGGGCAGCAATCCTCAGCTGACC<br>CCTCGGCAAGAGAACTGACGATTTCTGGATCCACGCT<br>AGCAATCAACCTCTGGATTACAAAATTTGTGAAAGAT<br>TGACTGGTATTCTTAACTATGTTGCTCCTTTTACGCTA<br>TGTGGATACGCTGCTTTAATGCCTTTGTATCATGCTAT<br>TGCTTCCCGTATGGCTTTCATTTTCTCCTCCTTGTATAA<br>ATCCTGGTTGCTGTCTCTTTATGAGGAGTTGTGGCCCG<br>TTGTCAGGCAACGTGGCGTGGTGTGCACTGTGTTTGCT<br>GACGCAACCCCCACTGGTTGGGGCATTGCCACCACCT<br>GTCAGCTCCTTTCCGGGACTTTCGCTTTCCCCCTCCCT<br>ATTGCCACGGCGGAACTCATCGCCGCCTGCCTTGCCC<br>GCTGCTGGACAGGGGCTCGGCTGTTGGGCACTGACAA<br>TTCCGTGGTGTTGTCGGGGAAATCATCGTCCTTTCCTT<br>GGCTGCTCGCCTGTGTTGCCACCTGGATTCTGCGCGG<br>GACGTCCTTCTGCTACGTCCCTTCGGCCCTCAATCCAG<br>CGGACCTTCCTTCCCGCGGCCTGCTGCCGGCTCTGCGG<br>CCTCTTCCGCGTCTTCGCCTTCGCCCTCAGACGAGTCG<br>GATCTCCCTTTGGGCCGCCTCCCCGCACCCAGCTTTCT<br>TGTACAAAGTGGGAATTCCTAGAGCTCGCTGATCAGC<br>CTCGACTGTGCCTTCTAGTTGCCAGCCATCTGTTGTTT<br>GCCCCTCCCCCGTGCCTTCCTTGACCCTGGAAGGTGCC<br>ACTCCCACTGTCCTTTCCTAATAAAATGAGGAAATTG<br>CATCGCATTGTCTGAGTAGGTGTCATTCTATTCTGGGG<br>GGTGGGGTGGGGCAGGACAGCAAGGGGGAGGATTGG<br>GAAGAGAATAGCAGGCATGCTGGGGAGGGCCGCAGG<br>AACCCCTAGTGATGGAGTTGGCCACTCCCTCTCTGCG<br>CGCTCGCTCGCTCACTGAGGCCGGGCGACCAAAGGTC<br>GCCCGACGCCCGGGCTTTGCCCGGGCGGCCTCAGTGA<br>GCGAGCGAGCGCGCAGCTGCCTGCAGGGGCGCCTGAT<br>GCGGTATTTTCTCCTTACGCATCTGTGCGGTATTTCAC<br>ACCGCATACGTCAAAGCAACCATAGTACGCGCCCTGT<br>AGCGGCGCATTAAGCGCGGCGGGTGTGGTGGTTACGC<br>GCAGCGTGACCGCTACACTTGCCAGCGCCTTAGCGCC<br>CGCTCCTTTCGCTTTCTTCCCTTCCTTTCTCGCCACGTT<br>CGCCGGCTTTCCCCGTCAAGCTCTAAATCGGGGGCTC<br>CCTTTAGGGTTCCGATTTAGTGCTTTACGGCACCTCGA<br>CCCCAAAAAACTTGATTTGGGTGATGGTTCACGTAGT

			GGGCCATCGCCCTGATAGACGGTTTTTCGCCCTTTGAC GTTGGAGTCCACGTTCTTTAATAGTGGACTCTTGTTCC AAACTGGAACAACACTCAACTCTATCTCGGGCTATTC TTTTGATTTATAAGGGATTTTGCCGATTTCGGTCTATT GGTTAAAAAATGAGCTGATTTAACAAAAATTTAACGC GAATTTTAACAAAATATTAACGTTTACAATTTTATGGT GCACTCTCAGTACAATCTGCTCTGATGCCGCATAGTTA AGCCAGCCCCGACACCCGCCAACACCCGCTGACGCGC CCTGACGGGCTTGTCTGCTCCCGGCATCCGCTTACAG ACAAGCTGTGACCGTCTCCGGGAGCTGCATGTGTCAG AGGTTTTCACCGTCATCACCGAAACGCGCGAGACGAA AGGGCCTCGTGATACGCCTATTTTTATAGGTTAATGTC ATGATAATAATGGTTTCTTAGACGTCCTGGCCCGTGTC TCAAAATCTCTGATGTTACATTGCACAAGATAAAAAT ATATCATCATGAACAATAAAACTGTCTGCTTACATAA ACAGTAATACAAGGGGTGTTATGAGCCATATTCAACG GGAAACGTCGAGGCCGCGATTAAATTCCAACATGGAT GCTGATTTATATGGGTATAAATGGGCTCGCGATAATG TCGGGCAATCAGGTGCGACAATCTATCGCTTGTATGG GAAGCCCGATGCGCCAGAGTTGTTTCTGAAACATGGC AAAGGTAGCGTTGCCAATGATGTTACAGATGAGATGG TCAGACTAAACTGGCTGACGGAATTTATGCCTCTTCC GACCATCAAGCATTTTATCCGTACTCCTGATGATGCAT GGTTACTCACCACTGCGATCCCCGGAAAAACAGCATT CCAGGTATTAGAAGAATATCCTGATTCAGGTGAAAAT ATTGTTGATGCGCTGGCAGTGTTCCTGCGCCGGTTGCA TTCGATTCCTGTTTGTAATTGTCCTTTTAACAGCGATC GCGTATTTCGTCTCGCTCAGGCGCAATCACGAATGAA TAACGGTTTGGTTGATGCGAGTGATTTTGATGACGAG CGTAATGGCTGGCCTGTTGAACAAGTCTGGAAAGAAA TGCATAAACTTTTGCCATTCTCACCGGATTCAGTCGTC ACTCATGGTGATTTCTCACTTGATAACCTTATTTTTGA CGAGGGGAAATTAATAGGTTGTATTGATGTTGGACGA GTCGGAATCGCAGACCGATACCAGGATCTTGCCATCC TATGGAACTGCCTCGGTGAGTTTTCTCCTTCATTACAG AAACGGCTTTTTCAAAAATATGGTATTGATAATCCTG ATATGAATAAATTGCAGTTTCATTTGATGCTCGATGA GTTTTTCTAATCAGAATTGGTTAATTGGTTGTAACACT GGCAGAGCATTACGCTGACTTGACGGGACGGCGCAA GCTCATGACCAAAATCCCTTAACGTGAGTTACGCGTG AAGATCCTTTTTGATAATCTCATGACCAAAATCCCTTA ACGTGAGTTTTCGTTCCACTGAGCGTCAGACCCCGTA GAAAAGATCAAAGGATCTTCTTGAGATCCTTTTTTTCT GCGCGTAATCTGCTGCTTGCAAACAAAAAAACCACCG CTACCAGCGGTGGTTTGTTTGCCGGATCAAGAGCTAC CAACTCTTTTTCCGAAGGTAACTGGCTTCAGCAGAGC GCAGATACCAAATACTGTTCTTCTAGTGTAGCCGTAG TTAGGCCACCACTTCAAGAACTCTGTAGCACCGCCTA CATACCTCGCTCTGCTAATCCTGTTACCAGTGGCTGCT GCCAGTGGCGATAAGTCGTGTCTTACCGGGTTGGACT CAAGACGATAGTTACCGGATAAGGCGCAGCGGTCGG

			GCTGAACGGGGGGTTCGTGCACACAGCCCAGCTTGGA<br>GCGAACGACCTACACCGAACTGAGATACCTACAGCGT<br>GAGCTATGAGAAAGCGCCACGCTTCCCGAAGGGAGA<br>AAGGCGGACAGGTATCCGGTAAGCGGCAGGGTCGGA<br>ACAGGAGAGCGCACGAGGGAGCTTCCAGGGGGAAAC<br>GCCTGGTATCTTTATAGTCCTGTCGGGTTTCGCCACCT<br>CTGACTTGAGCGTCGATTTTTGTGATGCTCGTCAGGGG<br>GGCGGAGCCTATGGAAAAACGCCAGCAACGCGGCCT<br>TTTTACGGTTCCTGGCCTTTTGCTGGCCTTTTGCTCAC<br>ATGT
4	AAVss-RK-KCNV2-WT	AAV2 5' ITR: 1-141 bp<br>RK: 169-788 bp<br>Kozak: 813-818 bp<br>KCNV2(WT): 819-2456 bp<br>WPRE(mut 6): 2457-3067 bp<br>BGH pA: 3122-3329 bp<br>AAV2 3' ITR: 3337-3477 bp	CCTGCAGGCAGCTGCGCGCTCGCTCGCTCACTGAGGC<br>CGCCCGGGCAAAGCCCGGGCGTCGGGCGACCTTTGGT<br>CGCCCGGCCTCAGTGAGCGAGCGAGCGCGCAGAGAG<br>GGAGTGGCCAACTCCATCACTAGGGGTTCCTTCTAGA<br>CAACTTTGTATAGAAAAGTTGTGTAGTTAATGATTAA<br>CCCGCCATGCTACTTATCTACGTACATTTATATTGGCT<br>CATGTCCAACATTACCGCCATGTTGACATTGATTATTG<br>ACTAGAATTCGCTAGCAAGATCCAAGCTCAGATCTCG<br>ATCGAGTTGGGCCCCAGAAGCCTGGTGGTTGTTTGTC<br>CTTCTCAGGGGAAAAGTGAGGCGGCCCCTTGGAGGAA<br>GGGGCCGGGCAGAATGATCTAATCGGATTCCAAGCAG<br>CTCAGGGGATTGTCTTTTTCTAGCACCTTCTTGCCACT<br>CCTAAGCGTCCTCCGTGACCCCGGCTGGGATTTAGCC<br>TGGTGCTGTGTCAGCCCCGGTCTCCCAGGGGCTTCCC<br>AGTGGTCCCCAGGAACCCTCGACAGGGCCCGGTCTCT<br>CTCGTCCAGCAAGGGCAGGGACGGGCCACAGGCCAA<br>GGGCCCTCGATCGAGGAACTGAAAAACCAGAAAGTT<br>AACTGGTAAGTTTAGTCTTTTTGTCTTTTATTTCAGGT<br>CCCGGATCCGGTGGTGGTGCAAATCAAAGAACTGCTC<br>CTCAGTGGATGTTGCCTTTACTTCTAGGCCTGTACGGA<br>AGTGTTACTTCTGCTCTAAAAGCTGCGGAATTGTACCC<br>GCGGCCGCCAAGTTTGTACAAAAAAGCAGGCTGCCAC<br>CATGCTCAAACAGAGTGAGAGGAGACGGTCCTGGAG<br>CTACAGGCCCTGGAACACGACGGAGAATGAGGGCAG<br>CCAACACCGCAGGAGCATTTGCTCCCTGGGTGCCCGT<br>TCCGGCTCCCAGGCCAGCATCCACGGCTGGACAGAGG<br>GCAACTATAACTACTACATCGAGGAAGACGAAGACG<br>GCGAGGAGGAGGACCAGTGGAAGGACGACCTGGCAG<br>AAGAGGACCAGCAGGCAGGGGAGGTCACCACCGCCA<br>AGCCCGAGGGCCCCAGCGACCCTCCGGCCCTGCTGTC<br>CACGCTGAATGTGAACGTGGGTGGCCACAGCTACCAG<br>CTGGACTACTGCGAGCTGGCCGGCTTCCCCAAGACGC<br>GCCTAGGTCGCCTGGCCACCTCCACCAGCCGCAGCCG<br>CCAGCTAAGCCTGTGCGACGACTACGAGGAGCAGAC<br>AGACGAATACTTCTTCGACCGCGACCCGGCCGTCTTC<br>CAGCTGGTCTACAATTTCTACCTGTCCGGGGTGCTGCT<br>GGTGCTCGACGGGCTGTGTCCGCGCCGCTTCCTGGAG<br>GAGCTGGGCTACTGGGGCGTGCGGCTCAAGTACACGC<br>CACGCTGCTGCCGCATCTGCTTCGAGGAGCGGCGCGA<br>CGAGCTGAGCGAACGGCTCAAGATCCAGCACGAGCT<br>GCGCGCGCAGGCGCAGGTCGAGGAGGCGGAGGAACT

			CTTCCGCGACATGCGCTTCTACGGCCCGCAGCGGCGC<br>CGCCTCTGGAACCTCATGGAGAAGCCATTCTCCTCGG<br>TGGCCGCCAAGGCCATCGGGGTGGCCTCCAGCACCTT<br>CGTGCTCGTCTCCGTGGTGGCGCTGGCGCTCAACACC<br>GTGGAGGAGATGCAGCAGCACTCGGGGCAGGGCGAG<br>GGCGGCCCAGACCTGCGGCCCATCCTGGAGCACGTGG<br>AGATGCTGTGCATGGGCTTCTTCACGCTCGAGTACCT<br>GCTGCGCCTAGCCTCCACGCCCGACCTGAGGCGCTTC<br>GCGCGCAGCGCCCTCAACCTGGTGGACCTGGTGGCCA<br>TCCTGCCGCTCTACCTTCAGCTGCTGCTCGAGTGCTTC<br>ACGGGCGAGGGCCACCAACGCGGCCAGACGGTGGGC<br>AGCGTGGGTAAGGTGGGTCAGGTGTTGCGCGTCATGC<br>GCCTCATGCGCATCTTCCGCATCCTCAAGCTGGCGCG<br>CCACTCCACCGGACTGCGTGCCTTCGGCTTCACGCTGC<br>GCCAGTGCTACCAGCAGGTGGGCTGCCTGCTGCTCTT<br>CATCGCCATGGGCATCTTCACTTTCTCTGCGGCTGTCT<br>ACTCTGTGGAGCACGATGTGCCCAGCACCAACTTCAC<br>TACCATCCCCCACTCCTGGTGGTGGGCCGCGGTGAGC<br>ATCTCCACCGTGGGCTACGGAGACATGTACCCAGAGA<br>CCCACCTGGGCAGGTTTTTTGCCTTCCTCTGCATTGCT<br>TTTGGGATCATTCTCAACGGGATGCCCATTTCCATCCT<br>CTACAACAAGTTTTCTGATTACTACAGCAAGCTGAAG<br>GCTTATGAGTATACCACCATACGCAGGGAGAGGGGA<br>GAGGTGAACTTCATGCAGAGAGCCAGAAAGAAGATA<br>GCTGAGTGTTTGCTTGGAAGCAACCCACAGCTCACCC<br>CAAGACAAGAGAATTAGCGATTTCTGGATCCACGCTA<br>GCAATCAACCTCTGGATTACAAAATTTGTGAAAGATT<br>GACTGGTATTCTTAACTATGTTGCTCCTTTTACGCTAT<br>GTGGATACGCTGCTTTAATGCCTTTGTATCATGCTATT<br>GCTTCCCGTATGGCTTTCATTTTCTCCTCCTTGTATAA<br>ATCCTGGTTGCTGTCTCTTTATGAGGAGTTGTGGCCCG<br>TTGTCAGGCAACGTGGCGTGGTGTGCACTGTGTTTGCT<br>GACGCAACCCCCACTGGTTGGGGCATTGCCACCACCT<br>GTCAGCTCCTTTCCGGGACTTTCGCTTTCCCCCTCCCT<br>ATTGCCACGGCGGAACTCATCGCCGCCTGCCTTGCCC<br>GCTGCTGGACAGGGGCTCGGCTGTTGGGCACTGACAA<br>TTCCGTGGTGTTGTCGGGGAAATCATCGTCCTTTCCTT<br>GGCTGCTCGCCTGTGTTGCCACCTGGATTCTGCGCGG<br>GACGTCCTTCTGCTACGTCCCTTCGGCCCTCAATCCAG<br>CGGACCTTCCTTCCCGCGGCCTGCTGCCGGCTCTGCGG<br>CCTCTTCCGCGTCTTCGCCTTCGCCCTCAGACGAGTCG<br>GATCTCCCTTTGGGCCGCCTCCCCGCACCCAGCTTTCT<br>TGTACAAAGTGGGAATTCCTAGAGCTCGCTGATCAGC<br>CTCGACTGTGCCTTCTAGTTGCCAGCCATCTGTTGTTT<br>GCCCCTCCCCCGTGCCTTCCTTGACCCTGGAAGGTGCC<br>ACTCCCACTGTCCTTTCCTAATAAAATGAGGAAATTG<br>CATCGCATTGTCTGAGTAGGTGTCATTCTATTCTGGGG<br>GGTGGGGTGGGGCAGGACAGCAAGGGGGAGGATTGG<br>GAAGAGAATAGCAGGCATGCTGGGGAGGGCCGCAGG<br>AACCCCTAGTGATGGAGTTGGCCACTCCCTCTCTGCG<br>CGCTCGCTCGCTCACTGAGGCCGGGCGACCAAAGGTC

			GCCCGACGCCCGGGCTTTGCCCGGGCGGCCTCAGTGA<br>GCGAGCGAGCGCGCAGCTGCCTGCAGGGGCGCCTGAT<br>GCGGTATTTTCTCCTTACGCATCTGTGCGGTATTTCAC<br>ACCGCATACGTCAAAGCAACCATAGTACGCGCCCTGT<br>AGCGGCGCATTAAGCGCGGCGGGTGTGGTGGTTACGC<br>GCAGCGTGACCGCTACACTTGCCAGCGCCTTAGCGCC<br>CGCTCCTTTCGCTTTCTTCCCTTCCTTTCTCGCCACGTT<br>CGCCGGCTTTCCCCGTCAAGCTCTAAATCGGGGGCTC<br>CCTTTAGGGTTCCGATTTAGTGCTTTACGGCACCTCGA<br>CCCCAAAAAACTTGATTTGGGTGATGGTTCACGTAGT<br>GGGCCATCGCCCTGATAGACGGTTTTTCGCCCTTTGAC<br>GTTGGAGTCCACGTTCTTTAATAGTGGACTCTTGTTCC<br>AAACTGGAACAACACTCAACTCTATCTCGGGCTATTC<br>TTTTGATTTATAAGGGATTTTGCCGATTTCGGTCTATT<br>GGTTAAAAAATGAGCTGATTTAACAAAAATTTAACGC<br>GAATTTTAACAAAATATTAACGTTTACAATTTTATGGT<br>GCACTCTCAGTACAATCTGCTCTGATGCCGCATAGTTA<br>AGCCAGCCCCGACACCCGCCAACACCCGCTGACGCGC<br>CCTGACGGGCTTGTCTGCTCCCGGCATCCGCTTACAG<br>ACAAGCTGTGACCGTCTCCGGGAGCTGCATGTGTCAG<br>AGGTTTTCACCGTCATCACCGAAACGCGCGAGACGAA<br>AGGGCCTCGTGATACGCCTATTTTTATAGGTTAATGTC<br>ATGATAATAATGGTTTCTTAGACGTCCTGGCCCGTGTC<br>TCAAAATCTCTGATGTTACATTGCACAAGATAAAAAT<br>ATATCATCATGAACAATAAAACTGTCTGCTTACATAA<br>ACAGTAATACAAGGGGTGTTATGAGCCATATTCAACG<br>GGAAACGTCGAGGCCGCGATTAAATTCCAACATGGAT<br>GCTGATTTATATGGGTATAAATGGGCTCGCGATAATG<br>TCGGGCAATCAGGTGCGACAATCTATCGCTTGTATGG<br>GAAGCCCGATGCGCCAGAGTTGTTTCTGAAACATGGC<br>AAAGGTAGCGTTGCCAATGATGTTACAGATGAGATGG<br>TCAGACTAAACTGGCTGACGGAATTTATGCCTCTTCC<br>GACCATCAAGCATTTTATCCGTACTCCTGATGATGCAT<br>GGTTACTCACCACTGCGATCCCCGGAAAAACAGCATT<br>CCAGGTATTAGAAGAATATCCTGATTCAGGTGAAAAT<br>ATTGTTGATGCGCTGGCAGTGTTCCTGCGCCGGTTGCA<br>TTCGATTCCTGTTTGTAATTGTCCTTTTAACAGCGATC<br>GCGTATTTCGTCTCGCTCAGGCGCAATCACGAATGAA<br>TAACGGTTTGGTTGATGCGAGTGATTTTGATGACGAG<br>CGTAATGGCTGGCCTGTTGAACAAGTCTGGAAAGAAA<br>TGCATAAACTTTTGCCATTCTCACCGGATTCAGTCGTC<br>ACTCATGGTGATTTCTCACTTGATAACCTTATTTTTGA<br>CGAGGGGAAATTAATAGGTTGTATTGATGTTGGACGA<br>GTCGGAATCGCAGACCGATACCAGGATCTTGCCATCC<br>TATGGAACTGCCTCGGTGAGTTTTCTCCTTCATTACAG<br>AAACGGCTTTTTCAAAAATATGGTATTGATAATCCTG<br>ATATGAATAAATTGCAGTTTCATTTGATGCTCGATGA<br>GTTTTTCTAATCAGAATTGGTTAATTGGTTGTAACACT<br>GGCAGAGCATTACGCTGACTTGACGGGACGGCGCAA<br>GCTCATGACCAAAATCCCTTAACGTGAGTTACGCGTG<br>AAGATCCTTTTTGATAATCTCATGACCAAAATCCCTTA

			ACGTGAGTTTTCGTTCCACTGAGCGTCAGACCCCGTA GAAAAGATCAAAGGATCTTCTTGAGATCCTTTTTTTCT GCGCGTAATCTGCTGCTTGCAAACAAAAAAACCACCG CTACCAGCGGTGGTTTGTTTGCCGGATCAAGAGCTAC CAACTCTTTTTCCGAAGGTAACTGGCTTCAGCAGAGC GCAGATACCAAATACTGTTCTTCTAGTGTAGCCGTAG TTAGGCCACCACTTCAAGAACTCTGTAGCACCGCCTA CATACCTCGCTCTGCTAATCCTGTTACCAGTGGCTGCT GCCAGTGGCGATAAGTCGTGTCTTACCGGGTTGGACT CAAGACGATAGTTACCGGATAAGGCGCAGCGGTCGG GCTGAACGGGGGGTTCGTGCACACAGCCCAGCTTGGA GCGAACGACCTACACCGAACTGAGATACCTACAGCGT GAGCTATGAGAAAGCGCCACGCTTCCCGAAGGGAGA AAGGCGGACAGGTATCCGGTAAGCGGCAGGGTCGGA ACAGGAGAGCGCACGAGGGAGCTTCCAGGGGGAAAC GCCTGGTATCTTTATAGTCCTGTCGGGTTTCGCCACCT CTGACTTGAGCGTCGATTTTTGTGATGCTCGTCAGGGG GGCGGAGCCTATGGAAAAACGCCAGCAACGCGGCCT TTTTACGGTTCCTGGCCTTTTGCTGGCCTTTTGCTCAC ATGT

Supplementary Table S11

Construct	Titre (VG/ml) Signagen
AAV5 CAG-KCNV2-WT	1.23E+13
AAV5 CAG-KCNV2-opt	1.44E+13
AAV5 RK-KCNV2-WT	1.10E+13
AAV5 RK-KCNV2-opt	2.30E+13
AAV7m8-CAG-KCNV2-WT	1.88E+13
AAV7m8-CAG-KCNV2-opt	1.09E+13
AAV7m8-RK-KCNV2-WT	1.52E+13
AAV7m8-RK-KCNV2-opt	2.04E+13
